# Attention-Enhanced YOLOv11 for Early Detection of Fungal-Induced Forest Tree Decline

**DOI:** 10.3390/plants15172609

**Published:** 2026-08-26

**Authors:** Farkhod Akhmedov, Doston Khasanov, Sarvarbek Sodikovich Yusupov, Oybek Usmankulovich Mallaev, Halimjon Ergashevich Khujamatov, Toshtemir Abdikhafizovich Khujakulov, Young Im Cho

**Affiliations:** 1 Department of Computer Engineering, Gachon University, Sujeong-gu, Seongnam-si 13120, Gyeonggi-do, Republic of Korea; farhodsam92@gmail.com; 2 Department of Data Communication Networks and Systems, Tashkent University of Information Technologies, Tashkent 100200, Uzbekistan; dhasanov@tuit.uz (D.K.); kh.khujamatov@tuit.uz (H.E.K.); 3 Department of Mechanical Engineering, Kimyo International University in Tashkent, Tashkent 100121, Uzbekistan; s.yusupov@kiut.uz; 4 Department of Digital Technologies, Alfraganus University, Yukori Karakamish Street 2a, Tashkent 100190, Uzbekistan; o.mallayev@afu.uz; 5 Faculty of Energy, National Research University “Tashkent Institute of Irrigation and Agricultural Mechanization Engineers Institute”, Tashkent 100000, Uzbekistan; 6 Department of Electronics and Instrumentation, Fergana State Technical University, Fergana 150107, Uzbekistan; 7 Department of Computer Engineering, University of Tashkent for Applied Sciences, Tashkent 100125, Uzbekistan; t.khujakulov@utas.uz

**Keywords:** fungi detection, tree health monitoring, object recognition, attention mechanisms, deep learning

## Abstract

Pathogenic fungi and their synergistic interactions with bark beetles, leading to vascular dysfunction, physiological stress, and eventual tree mortality, increasingly threaten forest ecosystems. Because fungal colonization often precedes visible macroscopic symptoms, early detection remains a critical yet challenging task in forest health monitoring. This study proposes a real-time deep learning-based object detection framework for identifying harmful fungi in proximity to host trees to support early intervention strategies. A custom dataset comprising 8900 images was constructed to represent two classes: Healthy and Unhealthy trees, where fungal presence is detected either directly on the tree or within its immediate ecological vicinity (e.g., near root systems). A fine-tuned YOLOv11 detection architecture is developed and augmented with a squeeze-and-excitation (SE)-like attention mechanism to enhance texture-sensitive feature representation. The model is trained and evaluated using precision, recall, F1-score and mean Average Precision (mAP). Experimental results demonstrate an overall mAP@0.5 of 0.825, with class-wise average precision values of 0.926 (Healthy) and 0.724 (Unhealthy). The Healthy class achieved classification accuracy of 0.92, while 0.71 of Unhealthy instances were correctly detected. F1-Confidence and recall-Confidence metrics indicate that optimal operational performance occurs within a confidence threshold range of 0.30–0.35, balancing false positives and false negatives. Despite the approximately balanced class distribution (50.6% Healthy and 49.4% Unhealthy), detection performance for the Unhealthy class was comparatively lower because of its greater intra-class variability, heterogeneous fungal appearance, and subtle visual manifestations. Findings demonstrate the feasibility of deploying real-time object detection models for early-stage fungal surveillance and highlight the importance of confidence calibration for operational disease monitoring systems.

## 1. Introduction

Fungi represent one of the most pervasive and destructive biological threats to global agriculture, food security, ecosystem stability, and public health. Pathogenic fungi are responsible for substantial crop losses worldwide, with estimates indicating that fungal diseases account for approximately 10–20 of global agriculture yield reductions annually (Fisher et al, 2012) [1]. In staple crops such as wheat, rice, maize, and potatoes, fungal infections can lead not only to reduced productivity but also to compromised food quality and safety. Beyond agricultural impacts, certain fungal species produce mycotoxins—secondary metabolites that pose serious health risks to humans and livestock. Mycotoxin contamination in food chains has been linked to immunosuppression, carcinogenicity, and acute toxicity (Bennett & Klicj, 2003) [2]. For example, aflatoxins produced by Aspergillus species are classified as Group 1 carcinogens by the World Health Organization. Consequently, early detection and monitoring of fungal growth are critical for mitigating public health risks and ensuring food safety. Environmental and climatic changes further exacerbate fungal proliferation and geographic spread. Increasing temperatures, humidity fluctuations, and altered precipitation patterns have been associated with expanded ranges of fungal pathogens and the emergence of new disease outbreaks (Bebber et al., 2013) [3]. These changes intensify the need for rapid, scalable, and automated detection systems capable of operating under variable field conditions. Traditionally, fungal detection has relied on manual visual inspection, microbiological culturing, and molecular diagnostic techniques. While laboratory-based approaches such as polymerase chain reaction (PCR) provide high specificity, they are time-consuming, require specialized equipment, and are impractical for large-scale field deployment (Lievens et al., 2006) [4]. Manual inspection, on the other hand, is labor-intensive and subject to observer bias, limiting its reliability and scalability. Datasets have played a critical role throughout the history of computer vision and image processing, driving research in diverse and challenging directions by providing standardized benchmarks for training and evaluating algorithms. Significant advancements in computer vision have been largely fueled by openly accessible datasets and well-defined evaluation protocols. In microscopic image analysis, datasets designed for nuclei segmentation and detection—structures that visually resemble fungal spores—have contributed to methodological developments in small-object detection. A large dataset comprising 29,000 images with annotated nuclear centers was introduced in [5], although precise boundary annotations were not fully available. Wienert et al. [6] later presented a dataset of histopathological images with detailed cellular and tissue boundary annotations, enabling more accurate segmentation tasks. Similarly, Gelasca et al. [7] produced a benchmark dataset for image segmentation and tracking across subcellular and tissue levels, incorporating ground-truth annotations to facilitate quantitative evaluation. Despite these advances, comparatively limited attention has been directed toward datasets and automated detection systems for ecological and agricultural fungal propagation, particularly in the context of insect–fungus interactions. In forest and agricultural ecosystems, certain beetle species act as biological vectors, transporting fungal spores and hyphae to healthy trees. These symbiotic or pathogenic relationships can initiate early-stage fungal colonization in bark tissues or surrounding soil environments. The transported spores germinate under favorable moisture and nutrient conditions, forming hyphal networks that expand within the soil matrix. Over time, these hyphae develop into mycelial structures and, in some cases, produce visible fruiting bodies (young mushrooms), potentially compromising plant health and ecosystem stability.

### Research Objective

**Figure 1:**
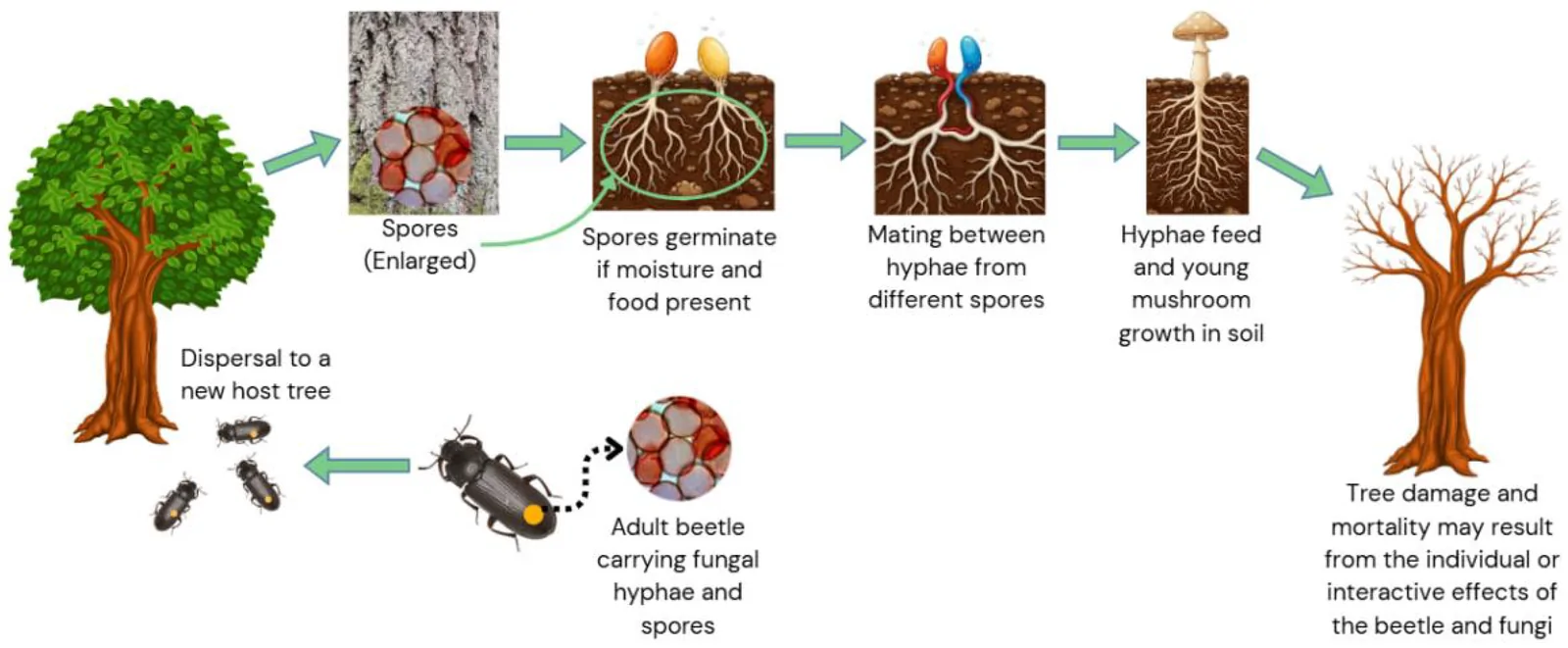
Schematic overview of fungus-associated tree decline and the interaction between fungal spores, host trees, and insect vectors. Fungal spores germinate under favorable environmental conditions, followed by hyphal development and growth within the host environment. Insect vectors may carry fungal hyphae and spores to new host trees, contributing to fungal dispersal and subsequent tree damage or mortality. The schematic components were adapted from publicly available sources and re-annotated and reorganized by the authors for illustrative purposes.

This study aims to develop a real-time object detection model capable of accurately identifying harmful fungi to support early intervention strategies. The figure schematically illustrates the ecological life cycle of a pathogenic fungus and its symbiotic association with bark beetles, culminating in host tree decline and mortality. The process begins with fungal spore production and dispersal from an infected host tree. These spores, shown in enlarged view, represent the primary reproductive units responsible for colonization. Upon dispersal to a new host—often mediated by adult beetles—the spores encounter suitable environmental conditions, including adequate moisture and nutrient availability, which trigger germination. Following germination, spores develop into filamentous hyphae that penetrate and colonize the woody substrate. When compatible hyphae originating from distinct spores interact, mating occurs, leading to the formation of a genetically stable mycelial network. In Figure 1, the visual elements were adapted from publicly available reference images and computationally pre-processed to isolate the relevant biological components. The resulting elements were subsequently integrated, arranged, and annotated by the authors using draw.io (diagrams.net) to illustrate the sequential stages and biological interactions associated with fungal infection.

This interconnected hyphal system expands within the vascular tissues of the host tree, extracting nutrients and disrupting water transport. The illustration further depicts early mushroom formation within the soil or host substrate, representing the reproductive stage that enables further spore dissemination and continuation of the life cycle. A critical component of this pathogenic cycle is the vectoring role of adult beetles. The figure shows beetles carrying fungal spores and hyphae exoskeletons or within specialized structures. As beetles disperse to new trees, they introduce fungal inoculum into fresh host tissues during bark penetration. This insect–fungus symbiosis amplifies disease spread, as the beetles facilitate fungal colonization while the fungus may weaken host defense, creating a mutually reinforcing pathogenic interaction. The final stage depicted illustrates tree damage and mortality resulting from either the independent effects of fungal infection and beetle infestation or their combined synergistic impact. Vascular occlusion, tissue necrosis, and systemic physiological stress ultimately compromise tree vitality, often leading to widespread forest decline. From a research perspective, this life-cycle framework underscores the importance of developing robust vision-based fungal detection systems, particularly for early-stage identification in or near apparently healthy trees [8,9,10].

The main contributions of this study are summarized as follows:

- **Development of a customized forest-health image dataset:** A domain-specific dataset comprising 8900 images was constructed for the detection of Healthy and Unhealthy tree conditions associated with visually observable fungal presence. The dataset incorporates real forest imagery, data annotation and augmentation, and alpha-compositing-based synthetic sample generation to increase the diversity of fungal appearances and environmental backgrounds.
- **Development of an attention-enhanced YOLOv11 framework:** A lightweight squeeze-and-excitation (SE)-inspired channel-attention mechanism was integrated at the beginning of the YOLOv11 neck, immediately after backbone feature extraction and before multi-scale feature fusion. The proposed mechanism recalibrates channel-wise feature responses to emphasize discriminative fungal-associated visual characteristics while suppressing less informative background features.
- **Comprehensive experimental and ablation evaluation:** The proposed framework was evaluated using precision, recall, F1-score, mAP@0.5, and mAP@0.5:0.95, together with class-wise and error analyses. Ablation experiments involving the baseline YOLOv11, CBAM-enhanced variant, and proposed SE-like configuration were conducted to assess the contribution of the attention mechanism to detection performance.
- **Evaluation of computational efficiency for practical deployment:** In addition to detection accuracy, the proposed model was evaluated in terms of parameter count, computational complexity, inference latency, throughput, and memory consumption. The resulting compact architecture demonstrates the potential of the proposed framework as an efficient visual screening tool for resource-constrained forest-health monitoring applications.

The remainder of this paper is organized as follows. Section 2 reviews related studies on computer-vision- and deep-learning-based approaches for plant disease, fungal infection, and forest-health monitoring, with particular emphasis on object detection and attention-based methods. Section 3 presents the proposed methodology, including dataset construction and annotation, synthetic data generation and augmentation, the YOLOv11 architecture, and the integration of the SE-like channel attention mechanism. Section 4 describes the experimental setup, training configuration, evaluation metrics, and quantitative and qualitative results, including detection performance, computational efficiency, and error analysis. Section 5 discusses the experimental findings, class-wise performance characteristics, ablation analysis, practical implications, and limitations of the proposed approach. Finally, Section 6 summarizes the main conclusions of the study and outlines directions for future research toward more robust and generalizable forest-health monitoring.

## 2. Related Works

Tree cover loss and forest degradation remain among the most pressing global environmental challenges of the 21st century. According to the World Resources Institute’s Global Forest Review, the world has lost hundreds of millions of hectares of tree cover since the turn of the century, with approximately 517 million hectares lost globally between 2001 and 2024, equivalent to about 13% of global tree cover in 2000. Annual rates of loss have also increased substantially over the same period, rising from over 13 million hectares per year in 2001 to nearly 30 million hectares per year by 2024. These figures underscore the magnitude of forest loss, driven by complex interactions of agricultural expansion, logging, wildlife, and other disturbances that reduce the extent and integrity of forest ecosystems [11]. The long-term consequences of continued tree cover reduction are significant for biodiversity, carbon sequestration, and ecosystem services. Permanent conversion of forested land to agriculture or infrastructure accounted for roughly 34% of global tree cover loss between 2001 and 2024, indicating a substantial fraction of loss that is unlikely to regenerate naturally [12]. Although many drivers of tree cover loss such as wildfire and forestry operations may be temporary, the cumulative degradation of forests can alter structural complexity and reduce resilience to subsequent stressors. Moreover, the loss of forest cover not only diminishes habitat for countless species but also affects below ground biotic interactions; for example, shifts in vegetation structure and land use have been shown to disrupt plant–soil symbiotic relationships and promote soil-borne pathogenic fungi at the expense of beneficial fungal symbionts [13]. Amid these large-scale trends, disease processes—including those mediated by pathogenic fungi—contribute to tree mortality and forest degradation across multiple biomass. Fungal pathogens such as Hymenoscyphus fraxineus have caused mortality rates of up 85% in ash tree plantations and woodlands in Europe, illustrating the capacity of fungal disease to effect substantial declines in tree populations [14,15,16]. Such pathogen-driven losses emphasize that forest decline is not only a product of direct anthropogenic land use change but also of biotic stressors that can spread rapidly once established. Several research efforts have explored fungal and plant disease detection using state-of-the-art (SOTA) methods. Mohanty et al. (2016) [17] demonstrated the effectiveness of D-CNNs for plant disease classification using leaf images, achieving high accuracy under controlled conditions. Researchers [17] investigated the efficacy of CNNs (AlexNet, GoogLeNet) on a large dataset of plant disease images, demonstrating classification accuracies exceeding 99% under controlled conditions. While CNN classification excels at identifying presence/absence of disease, its application to spatially localized detection of fungal structures in complex scenes is inherently limited. Standard CNN classification outputs a single label per image, making it unsuitable for tasks requiring localization of multiple lesions or fruiting bodies in forest imagery. This shortcoming motivates adoption of object detection frameworks capable of producing both class and bounding box predictions. Ferentinos (2018), [18] further evaluated CNN architectures for plant disease recognition across multiple species. More recently, object detection frameworks such as YOLO-based models [19] and Faster R-CNN [20] have been applied for fruiting body detection and wood-decay identification in forestry contexts. Transformer-based models [21] (e.g., DERT) have also been investigated for improved localization performance. Vision-based systems can analyze subtle bark discoloration, microstructural anomalies, early fruiting body formation, or beetle entry points that may not be readily apparent to a human observer. Detecting fungal presence in adjacent or visually healthy trees is especially critical, as asymptomatic hosts may already harbor latent infections that contribute to silent disease propagation. Early efforts in fungal and plant detection predominantly relied on classical image processing techniques that exploited handcrafted features to discriminate infected from healthy tissues. Such methods typically involve pre-processing (e.g., GLCM, LBP), color histograms, and shape descriptors, and subsequent classification using traditional machine learning algorithms (e.g., SVM, Random Forest). For instance, Barbedo et al. [22] utilized color and texture features for quantifying disease severity in foliar images, demonstrating that histogram thresholds and morphology could separate symptomatic from non-symptomatic regions. Similarly, Phadikar and Sil [23] applied Gabor filters and color segmentation to identify leaf blast symptoms, reporting moderate classification accuracy but strong sensitivity to lighting variation and background clutter. CNNs represents a paradigm shift in visual recognition by learning hierarchical representations directly from raw pixel data without manual feature engineering. In plant pathology, CNNs have been widely adopted for disease classification from leaf and stem images. Similarly, Too et al. [24] benchmarked multiple CNN (DenseNet, Inception, ResNet) across 10 fungal and viral plant disease datasets, noting that residual connections and dense arrangements improved classification robustness. However, most CNN-based classifiers operate at the image level—outputting a single label per sample—which restricts their applicability for localization of multiple symptomatic regions or object-level fungal detection. Table 1 below include reports of plant disease research works, proposed methods, key results and strength.

Recent advances in DL have substantially improved automated plant-disease recognition, particularly through object-detection frameworks capable of simultaneously classifying and localizing disease-associated visual features. One-stage detectors such as the YOLO family are widely adopted because of their favorable accuracy–speed trade-off, whereas two-stage architectures such as Faster R-CNN can provide stronger localization performance at the expense of a more complex inference pipeline. Recent work has therefore increasingly focused on lightweight backbones, attention mechanisms, and multi-scale feature fusion to improve discriminative representation while controlling computational cost. For example, Li et al. [25] proposed a lightweight locality-aware Faster R-CNN incorporating MobileNetV3X and coordinate attention, demonstrating substantial reductions in parameters and FLOPs while preserving competitive pest-detection performance across agricultural and forestry datasets. Attention-based feature enhancement is particularly relevant when disease-associated visual characteristics are small, low-contrast, or embedded within complex natural backgrounds. Zhang et al. [33] incorporated SENet channel attention into an improved YOLOv4 framework for UAV-based pine wilt disease detection and showed that both the use and placement of channel attention influenced detection performance, with the final architecture achieving an mAP of 79.91%. Du et al. [34] combined lightweight feature extraction, SimAM attention, and adaptive feature fusion for pine wilt disease detection, reporting an mAP of 95.64%, recall of 91.28%, and 95.70 FPS. Similarly, attention-enhanced YOLO models have achieved strong results for crop disease lesions; Zhu et al. [35] reported precision of 92.64%, recall of 93.28%, and AP of 96.17% for grape black-rot spot detection using an improved YOLOv8 model with efficient multi-scale attention. Nevertheless, these results are obtained from substantially different disease types, acquisition environments, and evaluation datasets and should therefore not be interpreted as direct head-to-head comparisons. A further distinction is required between visual disease screening and biological diagnosis of fungal colonization. Molecular studies have demonstrated that wood-decay fungi can be detected before measurable structural deterioration occurs. Jasalavich et al. [30] detected wood-decay basidiomycetes using PCR and subsequently identified taxa using restriction-fragment analysis, including detection before measurable wood weight loss. Gonthier et al. [29] further developed taxon-specific multiplex PCR assays capable of detecting approximately 1 pg of fungal target DNA, with even lower concentrations detectable using real-time PCR, and validated the method on 129 naturally infected samples. Spectroscopic approaches have likewise demonstrated the potential to distinguish fungal mycelium within wood; FTIR microscopy was used to localize and discriminate two wood-decaying fungal species within experimentally infected beech wood. These methods provide diagnostic specificity unavailable to conventional RGB imaging but require specialized instrumentation, sampling, or laboratory processing. In Table 2 we see analysis of fungal disease types and vision-based detection implications.

Accordingly, a key research gap lies between highly specific but resource-intensive fungal diagnostic techniques and scalable image-based monitoring. The present study addresses this gap by investigating an SE-like channel-attention-enhanced YOLOv11 framework for rapid, non-contact detection of externally observable fungal-associated indicators on or near trees. Unlike species-level molecular diagnosis, the proposed model performs binary Healthy/Unhealthy screening and should therefore be interpreted as a surveillance mechanism for identifying potentially at-risk trees requiring further assessment rather than as definitive identification of fungal species or latent internal infection.

Existing computer-vision studies demonstrate that DL can achieve strong performance in plant-disease recognition; however, the majority focus on crop leaves, fruits, or disease-specific symptoms rather than fungal structures occurring within heterogeneous forest scenes. Recent YOLO-based approaches have increasingly incorporated attention mechanisms, multi-scale feature fusion, and lightweight backbones to improve sensitivity to small or weakly expressed disease features. Nevertheless, reported performance values across studies should be interpreted cautiously because differences in target classes, image acquisition conditions, dataset size, train–test protocols, and metric definitions prevent direct numerical comparison. Furthermore, biological evidence indicates that fungal diseases exhibit highly heterogeneous external manifestations and that some root- and wood-decay pathogens may become established internally before visible symptoms appear. Therefore, image-based models should be regarded primarily as visual screening tools for observable fungal-associated cues rather than definitive diagnostic systems for latent infection.

Moreover, classification accuracy in laboratory datasets often declines markedly under field conditions due to background complexity and subtle early infection cues. Faster R-CNN introduced a two-stage detection paradigm that combine region proposal networks with deep feature extraction. DL models for plant disease detection achieve up to 100% accuracy in controlled environments but face performance drops in real-world scenarios, where optimized architectures like Faster R-CNN and YOLO still maintain over 90% mAP. Designed to mitigate USD 220 billion in annual global crop losses, these technologies, including YOLOv5s, achieve 0.915 mAP at 7.72 ms inference speeds, although bridging the “lab-to-field” gap with robust, multi-stage field datasets remains a challenge for widespread adoption. For more details, see Zhou et al. [26]. The research [37] optimized pan-fungal primer sets targeting the highly conserved ribosomal RNA (rDNA) operon—specifically focusing on the internal transcribed spacer regions and the 18S/28S subunits. Comparative evaluations identified the FF2/FR1 primer combination as a premier candidate, capable of robustly amplifying over two dozen major indoor fungal genera (including Aspergillus, Penicillium, Cladosporium, and Stachybotrys) while entirely eliminating cross-reactivity with background mammalian (human, murine), bacterial, or plant pollen DNA.

The YOLO family of detectors has become a popular choice for real-time object detection [38] due to its single-stage end-to-end inference. Fu et al. [39] applied YOLOv3 to detect anthracnose lesions on mango fruit, reporting real-time performance with mAP > 0.78. Similarly, Li et al. [40] demonstrated the efficacy of YOLOv5 in detecting powdery mildew on grape leaves, with high precision and frames per second (FPS) suitable for mobile deployment. In forest pathology research, Feng et al. [41] utilized YOLOv8 to detect needle blight and cankers in pine imagery, achieving an mAP of 0.77 with inference speeds exceeding 30 FPS on a single GPU. YOLO models share the advantage of balancing detection accuracy and inference speed, particularly beneficial for large-scale aerial surveys. However, YOLO’s grid-based prediction mechanism can struggle with very small lesions or low-contrast fungal structures, often requiring extensive data augmentation or architectural enhancements. In this study, we have adjusted YOLO architecture according to our research object, and in the next section, we will discuss our attention layer. EfficientDet in fungal and pathogen detection is also a common approach that integrates compound scaling with a Bi-FPN architecture to balance accuracy and efficiency across multiple computational budgets. Despite these advantages, EfficientDet models typically incur higher computationally costs than lightweight YOLO variants, posing challenges in resource-constrained environments such as edge deployments or UAV platforms. Therefore, the primary research objective in this context is to design and optimize automated image-based detection framework capable of identifying early fungal indicators with high sensitivity and specificity.

## 3. Materials and Methods

**Figure 2:**
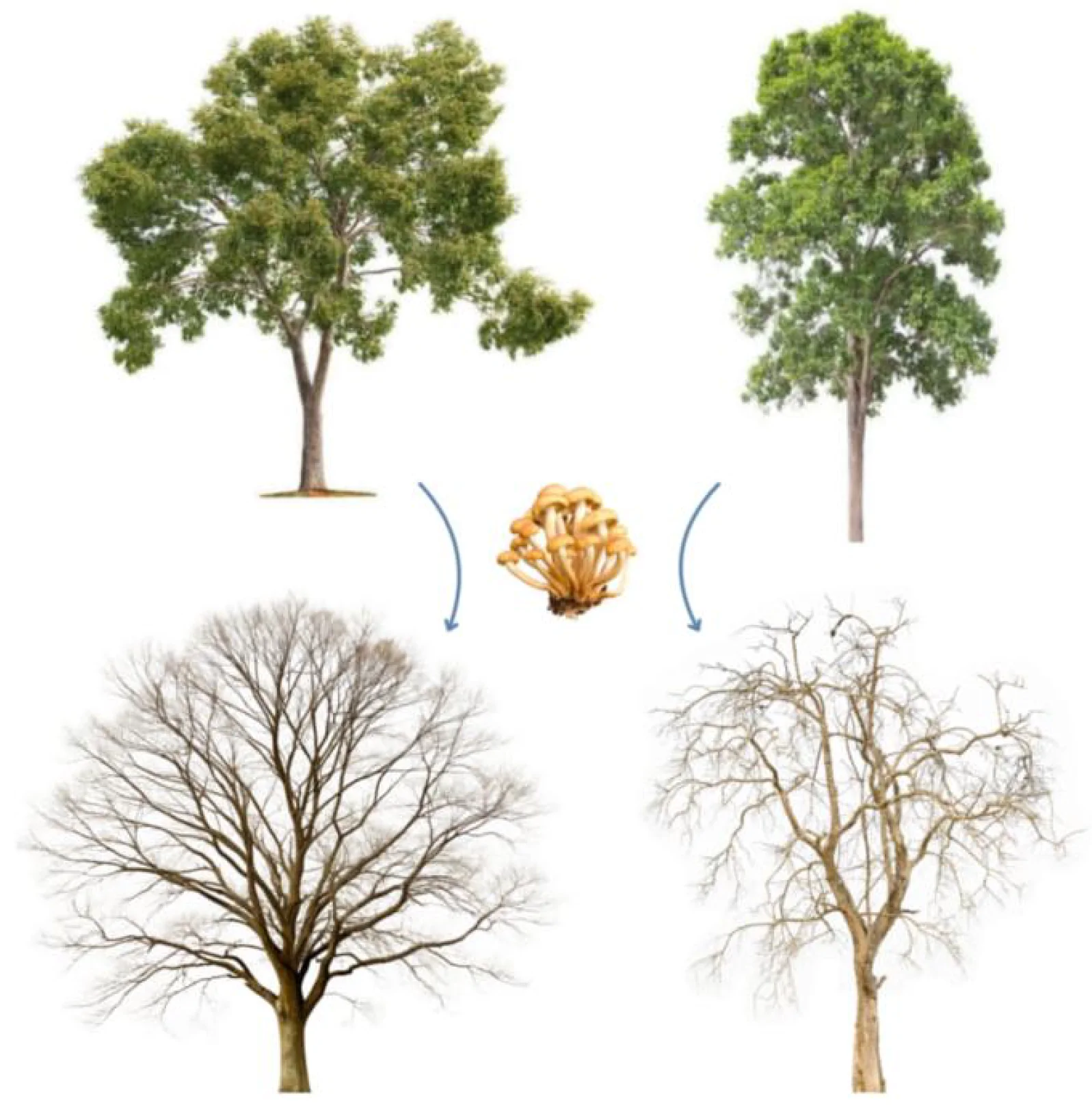
Fungi effect example.

Traditional approaches to monitoring tree health and disease incidence—indicating field-based surveys and manual visual inspection—face significant limitations, particularly at landscape and continental scales. Manual inspection is inherently labor-intensive, time-consuming, and subject to observer bias, making it impractical for frequent monitoring over large and remote areas. In heterogeneous forest ecosystems, early symptoms of pathogen colonization, such as subtle hyphal development in soil or initial canopy discoloration, can be easily overlooked or misclassified during routine surveys. Furthermore, logistical constraints limit the spatial resolution and temporal frequently of field assessments, delaying responses to rapidly spreading threats. These limitations impede timely intervention, allowing disease and associated tree loss to propagate unchecked. The increasing complexity and scale of tree health challenges have therefore catalyzed interest in automated detection systems that leverage advances in imaging and machine learning. Early detection of such fungal development is crucial for preventing large-scale tree infections and forest degradation. However, the visual characteristics of early-stage hyphal growth present substantial challenges for automated analysis. Hyphae are thin, filamentous structures that often exhibit low contrast with soil backgrounds and may be partially occluded by organic debris. Furthermore, morphological similarities between fungal spores, soil particles, and other objects require detection and segmentation models capable of handling small objects, high intra-class variability, and complex environmental noise. In Figure 2, we provide a general example of of the effects of fungal infection on a healthy tree.

From a computer vision perspective, this problem intersects with several challenging research areas, including small-object detection, fine-grained classification, multi-scale feature representation, and temporal growth modeling. Unlike conventional plant disease detection, which often focuses on visible leaf lesions, the present research emphasizes the detection of early subterranean fungal development initiated by beetle-mediated transmission. This shift toward early-stage detection introduces additional complexity due to limited visual cues and dynamic growth patterns.

**Figure 3:**
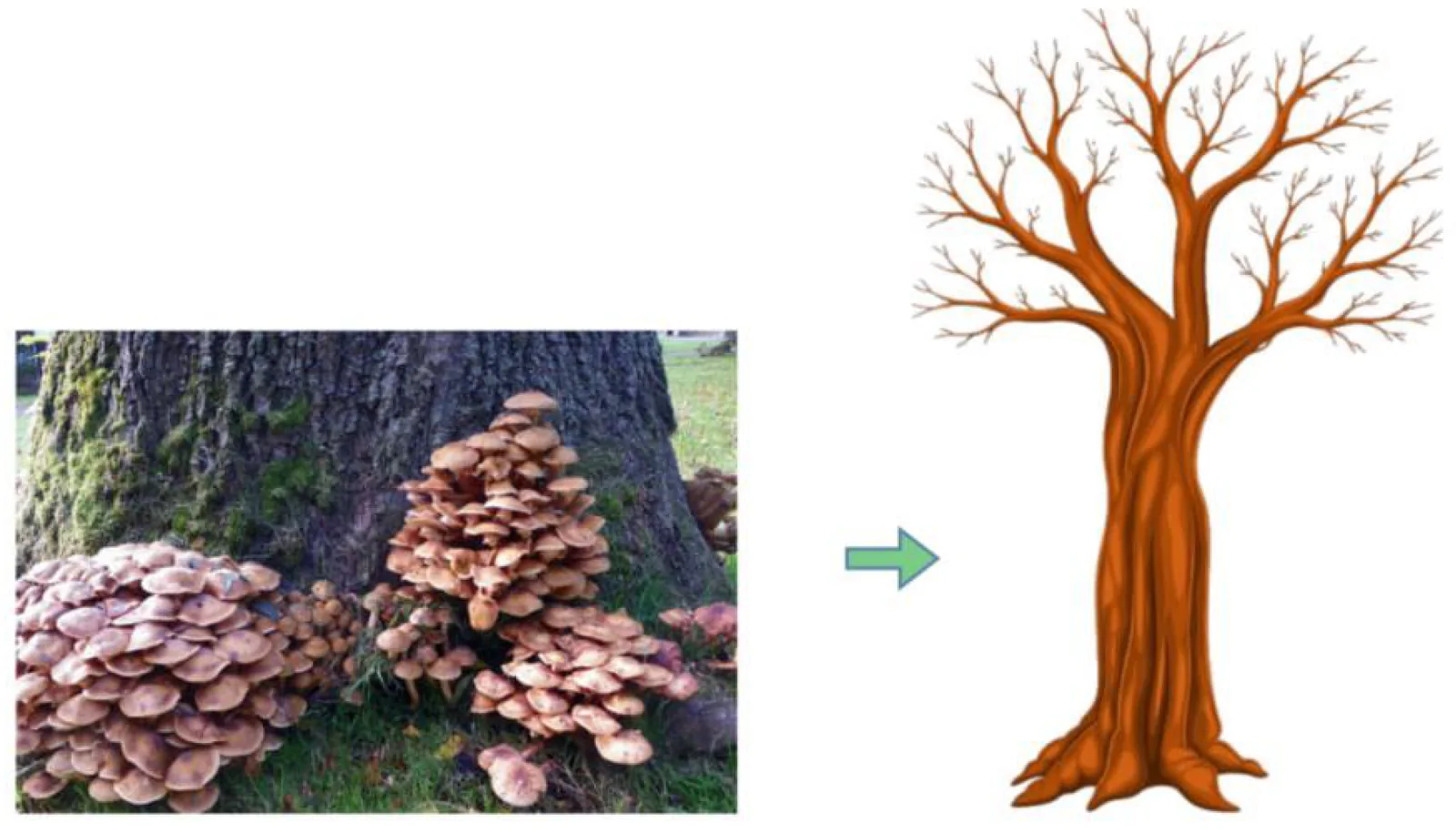
Left-side image from real life located nearby a tree root. Right-side tree image is an illustrative example of a dried tree.

Figure 3 below conceptually illustrates the progressive impact of fungal colonization on tree vitality, transforming from a physiologically healthy state to severe decline and eventually mortality. The upper row depicts two apparently healthy trees characterized by dense foliage, intact canopy architecture, and normal structural integrity. The central fungal cluster symbolizes the latent or emerging presence of a pathogenic fungus within or around the host system. The lower row demonstrates the outcome of sustained fungal infection: extensive canopy loss, branch dieback, vascular dysfunction, and structural degradation, ultimately culminating in tree death [42]. This visual representation encapsulates the pathogenic continuum in which fungal establishment precedes visible morphological deterioration.

From a biological perspective, fungal pathogens infiltrate host trees though wounds, root systems, or vector organisms (e.g., bark beetles), establishing mycelial networks that colonize xylem and phloem tissues [43]. Once established, hyphal expansion disrupts vascular conductivity, impedes water and nutrient transport, and induces host defense responses that may further occlude conductive tissues [44]. Let $V_{t}$ denote vascular volume conductivity at time t. Progressive fungal colonization can be conceptualized as a decay process:

(1) $$V_{t}=V_{0}e^{-\Delta t}$$

where $V_{o}$ is the initial conductivity and Δ represents the rate of fungal-induced vascular degradation. As $V_{t}$ → 0, physiological stress intensifies, leading to canopy thinning, chlorosis, necrosis, and eventually, structural collapse, as illustrated in the image above (Figure 3). Importantly, fungal colonization may remain asymptomatic during early stages, meaning that visually healthy trees may already harbor pathogenic activity. This latent phase underscores the critical importance of computer vision-based detection systems for early fungal identification, particularly under or near trees that appear healthy. Early detection models aim to identify subtle visual indicators—such as minor bark discoloration, micro-fruiting bodies, early canopy stress signatures, or structural anomalies—before irreversible vascular damage occurs. Formally, a vision-based detection framework learns a mapping:

(2) $$f_{\theta }:I\to \left\{y_{\mathrm{class}},b\right\},$$

where I is the input image, $y_{\mathrm{class}}\in \left\{\mathrm{healthy},\mathrm{infected}\right\}$, and b represents localized fungal regions. Advanced object detection architectures (e.g., YOLO, Faster R-CNN, DETR) optimize joint classification and localization objectives:

(3) $$L=\lambda_{\mathrm{cls}}L_{\mathrm{CE}}+\lambda_{\mathrm{reg}}L_{\mathrm{IoU}}$$

thereby enabling spatially explicit fungal growth identification. Deploying such systems in forestry monitoring can facilitate proactive intervention, reduce pathogen spread, and prevent large-scale ecological and economic loss.

### Dataset Preparation

**Figure 4:**
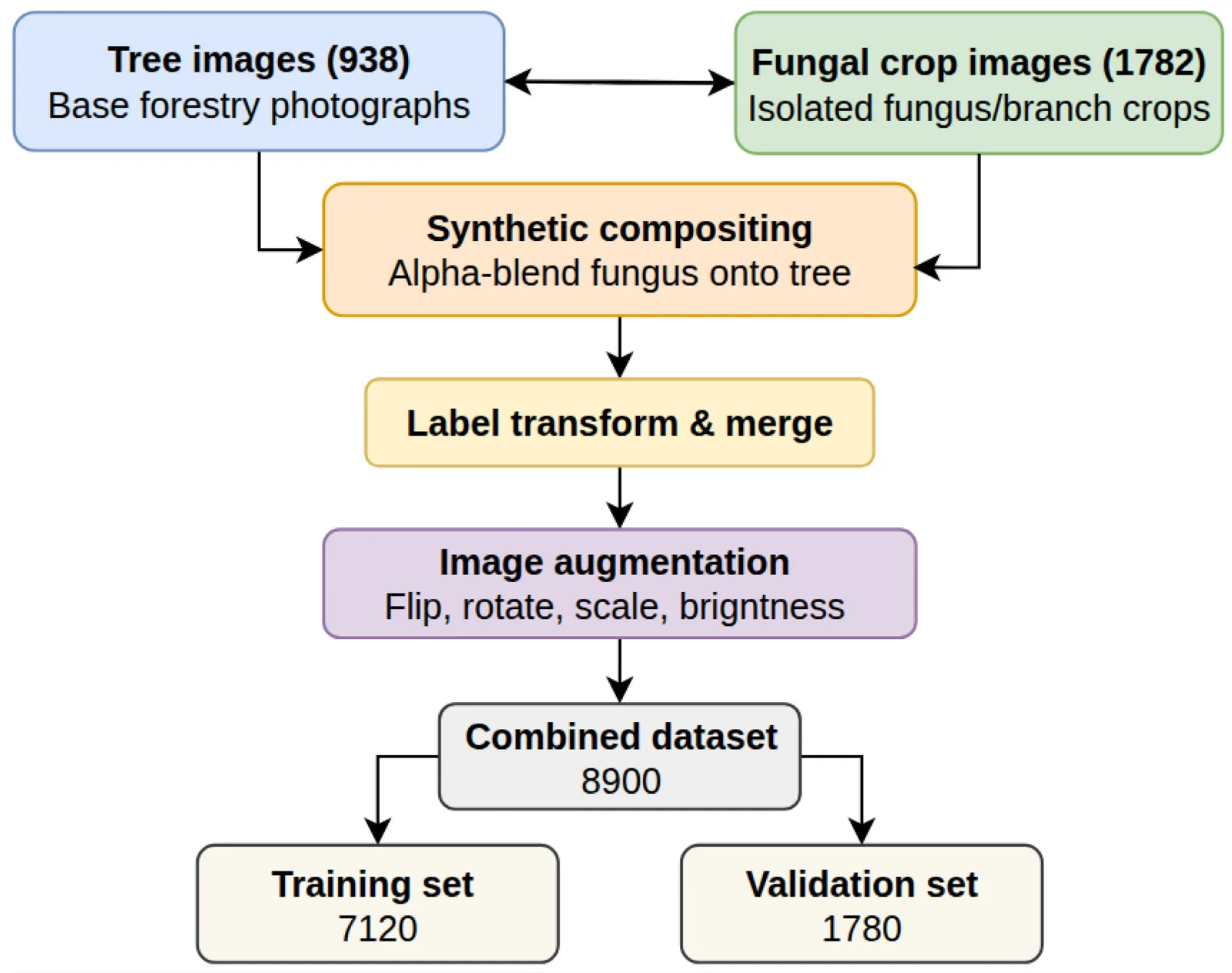
Dataset preparation process and train–validation splitting pipeline.

Figure 4 illustrates the dataset construction pipeline. A total of 938 base tree images and 1782 fungal crop images were used for synthetic compositing through alpha blending.

To train the proposed model for the visual detection of fungus-associated tree-health conditions, a customized image dataset was constructed using tree images representing both Healthy and Unhealthy conditions. Healthy samples were selected from images in which no visually observable fungal structures were present on the tree trunk, root region, or immediate surrounding area. In contrast, Unhealthy samples were defined based on the presence of externally visible fungal structures located either directly on the tree or within its immediate vicinity. This categorization was designed to enable the model to learn spatial and visual associations between observable fungal presence and tree-health condition.

**Figure 5:**
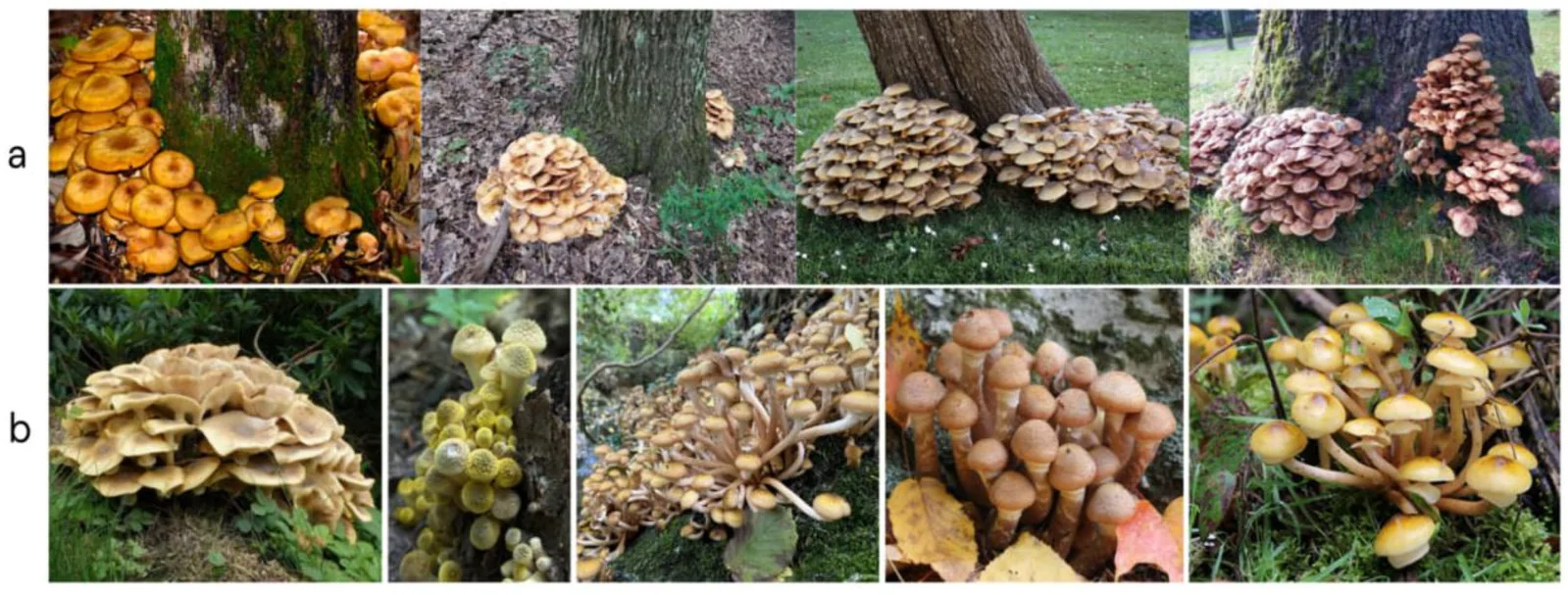
Unhealthy class example images. Fungi location is in immediate surroundings of tree—subsection (**a**). In (**b**), colonization of fungi is depicted.

Accordingly, the two target categories were defined as follows:

- **Healthy**—trees showing no visually observable fungal presence on the trunk, root region, or immediate surrounding area.
- **Unhealthy**—trees associated with visually observable fungal growth located directly on the trunk, around the root region, or in close proximity to the tree, as illustrated in Figure 5a,b.

The distinction between the two classes is therefore based on externally observable fungus-associated visual cues rather than laboratory-confirmed fungal infection or species-level identification. This formulation allows the proposed framework to serve as a non-contact visual screening approach for identifying potentially compromised trees and localizing visible fungal-associated regions in forest environments.

Even when structural damage is not yet visible, such trees are labeled as Unhealthy because fungal presence indicates an infectious and non-healthy ecological state.

Table 3 summarizes the dataset construction and preparation process, including data sources, class distribution, train–validation split, annotation procedure, synthetic compositing, and offline augmentation. The final dataset comprises 8900 RGB images, with 7120 used for training and 1780 for validation.

**Figure 6:**
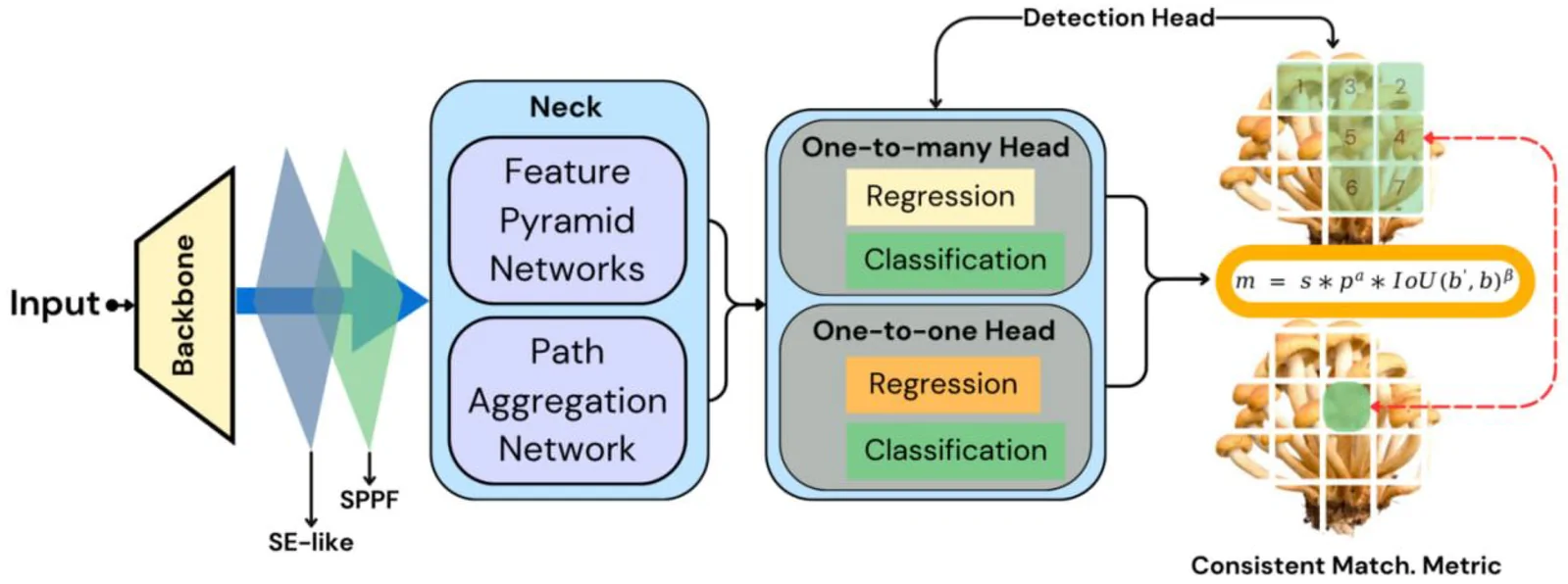
Fungi detection model architecture, adding SE-like in the beginning of neck of YOLO architecture.

Figure 6 illustrates the architecture of the proposed SE-like attention-enhanced YOLOv11 framework. Input images are processed by the backbone, followed by SE-like channel attention for feature recalibration. The extracted features are subsequently fused through the neck using feature pyramid and path aggregation networks. The detection head employs one-to-many and one-to-one branches for regression and classification, with a consistent matching metric used to associate predictions with ground-truth targets.

**Figure 7:**
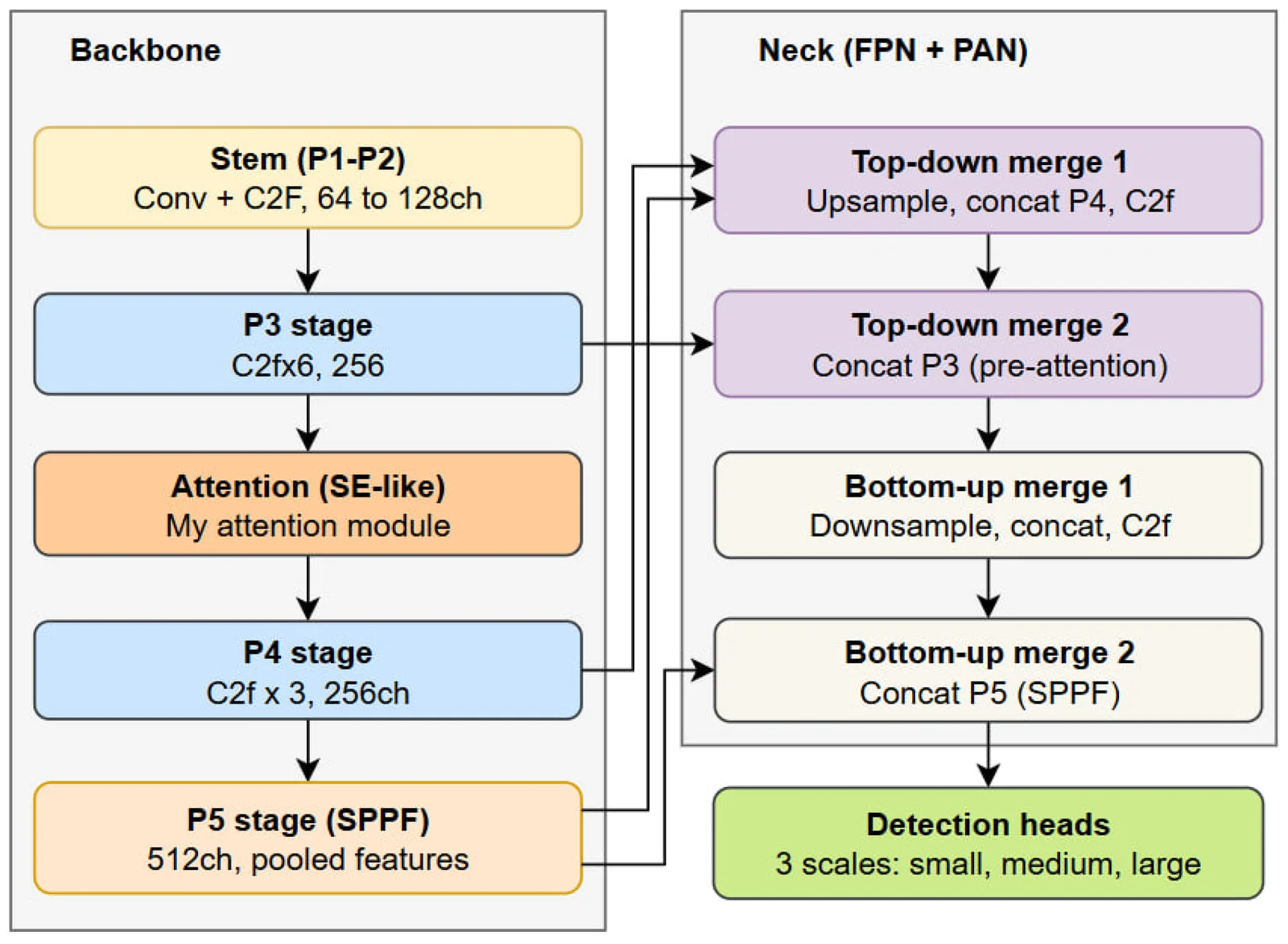
Fungi detection model architecture.

Figure 7 illustrates the detailed architecture and placement of the proposed SE-like attention mechanism within the YOLO-based feature extraction and fusion pipeline. The backbone progressively extracts hierarchical representations through the stem and P3, P4, and P5 stages, with the P5 stage incorporating spatial pyramid pooling fast (SPPF) for high-level feature aggregation.

The proposed SE-like attention module is introduced after the P3 feature-extraction stage and before the subsequent P4 stage, enabling channel-wise recalibration of intermediate features. The resulting multi-scale backbone features are subsequently propagated to the neck, where Feature Pyramid Network (FPN) and Path Aggregation Network (PAN) operations perform top-down and bottom-up feature fusion. Finally, the fused representations are forwarded to detection heads operating at three scales to support the localization and classification of targets with different spatial sizes. This architecture enables attention-guided feature refinement while preserving multi-scale information throughout the detection pipeline.

The input image is denoted as $I\in R^{H\times W\times 3}$. The backbone network β(·) extracts hierarchical feature representations:

(4) $${\left\{F_{l}\right\}}_{l=1}^{L}=\beta \left(I\right),$$

where $F_{l}\in R^{H_{l}\times W_{l}\times C_{l}}$ represents the feature map at scale *l*. The backbone integrates channel attention in an SE-like (Squeeze-and-Excitation) mechanism, which adaptively recalibrates channel response. Formally, for a feature tensor F, global average pooling generates a channel descriptor:

(5) $$z_{c}=\frac{1}{HW}\sum_{i=1}^{H}\sum_{j=1}^{W}F_{i,j,c},$$

where δ(·) denotes a non-linear activation (e.g., ReLU), σ(·) is the sigmoid function, and $W_{1}$, $W_{2}$ are learnable parameters. The recalibrated feature map is obtained via channel-wise multiplication:

(6) $${\tilde{F}}_{i,j,c}=s_{c}\cdot F_{i,j,c}$$

In addition, Spatial Pyramid Pooling Fast (SPPF) module expands the receptive field by aggregating multi-scale contextual information using parallel pooling operations, effectively enriching semantic representation without increasing computational burden significantly. The neck comprises a Feature Pyramid Network (FPN) and a Path Aggregation Network (PAN), collectively denoted as N(·). The FPN performs top-down lateral fusion:

(7) $$P_{l}=⌀\left(F_{l}\right)+\mathrm{Upsample}\left(P_{l+1}\right),$$

where ⌀(·) is a 1×1 convolutional for channel alignment. The PAN enhances bottom-up information flow:

(8) $$Q_{l}=\vartheta \left(P_{l}\right)+\mathrm{Downsample}\left(Q_{l-1}\right),$$

producing a set of multi-scale aggregated feature $\left\{Q_{l}\right\}$ that encode both high-level semantics and fine-grained localization cues.

The detection head consists of two parallel branches: a one-to-many head and a one-to-one head. For each spatial location *k* in feature map $Q_{l}$, the model predicts a bounding box regression vector ${\hat{b}}_{k}=\left(x,y,w,h\right)$ and class probabilities ${\hat{p}}_{k}\in {\left[0,1\right]}^{C}$.

The one-to-many head follows dense assignment, where multiple candidate predictions may correspond to a single ground-truth object. The loss for this branch can be formulated as

(9) $$\lambda_{\mathrm{OTO}}=\sum_{(k,g)\in M}\left[\tau_{\mathrm{cls}}\lambda_{\mathrm{CE}}\left({\hat{p}}_{k},p_{g}\right)+\tau_{\mathrm{cls}}\lambda_{\mathrm{IoU}}\left({\hat{b}}_{k},b_{g}\right)\right]$$

A consistent matching metric is introduced to unify classification confidence, localization quality, and spatial overlap. The matching score is defined as:

(10) $$m=s\cdot p^{\alpha }\cdot \mathrm{IoU}{\left({\hat{b}}_{k},b_{g}\right)}^{\beta },$$

where:

- *s* denotes the objectness score,
- *p* is the predicted class probability,
- $\mathrm{IoU}({\hat{b}}_{k},b_{g})$ measures the overlap between predicted and ground-truth boxes,
- α and β are hyperparameters balancing classification and localization contributions.

This formulation ensures that assignment and optimization are jointly influenced by both semantic confidence and geometric alignment, mitigating inconsistencies between classification and regression branches.

## 4. Experimental Results and Analysis

### 4.1. Model Training

The experiments were implemented using the Ultralytics YOLO framework with PyTorch 2.11.0. Model training and validation were performed on an NVIDIA GeForce RTX 4090 GPU using an input resolution of 640×640 pixels. Computational performance for CPU-based deployment was additionally evaluated on an AMD Ryzen Threadripper 1950X (made in New York, NY, USA) processor. The GPU-based training environment and CPU-based inference benchmark are reported separately to distinguish model training performance from deployment-oriented computational evaluation.

The custom dataset consisted of 8900 images and was divided into training and validation subsets using an 80:20 stratified split, ensuring that the relative proportions of the Healthy and Unhealthy classes were preserved across both subsets. The class-wise distribution of the dataset and the corresponding image parameters are presented in Table 3.

The model was trained for 50 epochs with a batch size of 8 and an input resolution of 640×640 pixels. The Ultralytics automatic optimizer-selection setting was used, with an initial learning-rate parameter ($lr_{0}$) of 0.01, a final learning-rate factor (lrf) of 0.01, momentum of 0.937, and weight decay of 0.0005. A fixed random seed of 0 was used for the reported training run. To improve the robustness of the proposed model, several standard data augmentation techniques were applied during training, including random horizontal flipping, mosaic augmentation, HSV color-space perturbation, random scaling, and translation. These augmentations increased the diversity of the training samples and enhanced the model’s ability to generalize to varying environmental conditions. The proposed squeeze-and-excitation (SE)-inspired attention module was integrated at the beginning of the neck network, immediately after feature extraction by the backbone and before multi-scale feature fusion. The attention module was trained jointly with the backbone, neck, and detection head in an end-to-end manner without requiring a separate pre-training stage. This enables the network to perform channel-wise feature recalibration while preserving the computational efficiency of the original YOLOv11 architecture. An SE-like channel-attention mechanism was selected because the visual characteristics relevant to fungal detection are frequently represented by subtle differences in texture, color, and local appearance that may be difficult to distinguish from tree bark, soil, leaves, and other organic background structures. Rather than applying additional spatial processing across the entire feature map, the SE-like mechanism recalibrates channel responses according to their global importance, strengthening feature channels associated with discriminative fungal characteristics while suppressing less informative responses. This design was also selected because of its relatively lightweight computational structure, which is compatible with the real-time objective of the proposed YOLOv11 framework (Table 4 ).

The attention-enhanced YOLOv11 model was trained using the Ultralytics YOLO detection framework. The custom model configuration was defined in my_yolo_attention.yaml, where an attention module was inserted into the backbone feature extraction stage. Training was performed using 640 × 640 pixel input images, a batch size of 8, and 50 epochs. The pretrained setting was enabled, automatic optimizer selection was used, and training was conducted on GPU device 0. The initial learning rate was 0.01, with a final learning-rate factor of 0.01, momentum of 0.937, and weight decay of 0.0005. Warm-up training was applied for 3 epochs, with warm-up momentum of 0.8 and warm-up bias learning rate of 0.1. The bounding-box, classification, and DFL loss gains were set to 7.5, 0.5, and 1.5, respectively. Data augmentation included HSV adjustment, image translation, scaling, horizontal flipping, mosaic augmentation, RandAugment, and random erasing. Validation was performed using an IoU threshold of 0.7 and a maximum detection number of 300.

### 4.2. Evaluation Metrics

In this study, definitions of the evaluation metrics derived from the confusion matrix are presented in Table 5. These metrics play a fundamental role in quantifying the discriminative capability of the proposed object detection model in distinguishing between Healthy and Unhealthy trees.

Precision, recall, F1-score, average precision (AP), and mean average precision (mAP) were employed to quantitatively evaluate the detection performance of the proposed framework. In addition, Intersection over Union (IoU) was used to determine the spatial agreement between predicted and ground-truth bounding boxes. These metrics jointly characterize the model’s detection reliability, sensitivity to fungus-associated targets, localization quality, and overall performance across the two target classes.

Precision measures the proportion of predicted positive detections that are correct and is defined as

(11) $$\mathrm{Precision}=\frac{T_{P}}{T_{P}+F_{P}},$$

where $T_{P}$ and $F_{P}$ denote the numbers of true-positive and false-positive detections, respectively. A high precision indicates that the model generates relatively few incorrect positive detections.

Recall measures the proportion of ground-truth positive instances that are successfully detected by the model and is expressed as

(12) $$\mathrm{Recall}=\frac{T_{P}}{T_{P}+F_{N}},$$

where $F_{N}$ represents false-negative detections. Recall is particularly important in the present application because missed fungus-associated targets may reduce the effectiveness of visual tree-health screening.

The F1-score represents the harmonic mean of precision and recall and provides a balanced measure when both false-positive and false-negative detections are important:

(13) $$F_{1}=2\times \frac{\mathrm{Precision}\times \mathrm{Recall}}{\mathrm{Precision}+\mathrm{Recall}}.$$

The spatial correspondence between a predicted bounding box $B_{p}$ and its corresponding ground-truth bounding box $B_{gt}$ was evaluated using Intersection over Union (IoU), defined as

(14) $$\mathrm{IoU}=\frac{|B_{p}\cap B_{gt}|}{|B_{p}\cup B_{gt}|},$$

where $|B_{p}\cap B_{gt}|$ denotes the intersection area and $|B_{p}\cup B_{gt}|$ denotes the union area of the predicted and ground-truth bounding boxes. A larger IoU indicates greater spatial agreement between the predicted and annotated target regions.

Average Precision (AP) summarizes the precision–recall relationship for an individual class and can be expressed as the area under the precision–recall curve:

(15) $$AP_{c}=\int_{0}^{1}P_{c}\left(R\right)\;dR,$$

where $P_{c}\left(R\right)$ denotes precision as a function of recall for class *c*. In this study, class-wise AP values are reported separately for the Healthy and Unhealthy target categories.

Mean Average Precision (mAP) represents the mean AP across all *C* target classes:

(16) $$mAP=\frac{1}{C}\sum_{c=1}^{C}AP_{c},$$

where C=2 in the present study, corresponding to the Healthy and Unhealthy classes. Two mAP measures were considered. mAP@0.5 evaluates AP using an IoU threshold of 0.50, whereas mAP@0.5:0.95 averages AP over IoU thresholds ranging from 0.50 to 0.95 in increments of 0.05. The latter provides a more stringent assessment of both detection and localization performance.

Table 6 summarizes the overall detection performance of the proposed SE-like YOLOv11 model. The model achieved a precision of 0.8461, indicating that a high proportion of the predicted detections were correct, while the recall of 0.7354 shows that the model successfully identified approximately 73.5% of the relevant instances. The resulting F1-score of 0.7869 reflects a reasonable balance between precision and recall. In terms of detection accuracy, the model obtained an mAP@0.5 of 0.8266, demonstrating strong localization and classification performance at an IoU threshold of 0.5. However, the mAP@0.5:0.95 decreased to 0.4739 when evaluated across progressively stricter IoU thresholds. This difference indicates that although the model reliably detects fungal-associated tree-health regions under the standard IoU criterion, achieving highly precise bounding-box localization becomes more challenging under stricter overlap requirements. Overall, the results demonstrate effective detection performance while also highlighting opportunities to further improve localization precision and recall.

**Figure 8:**
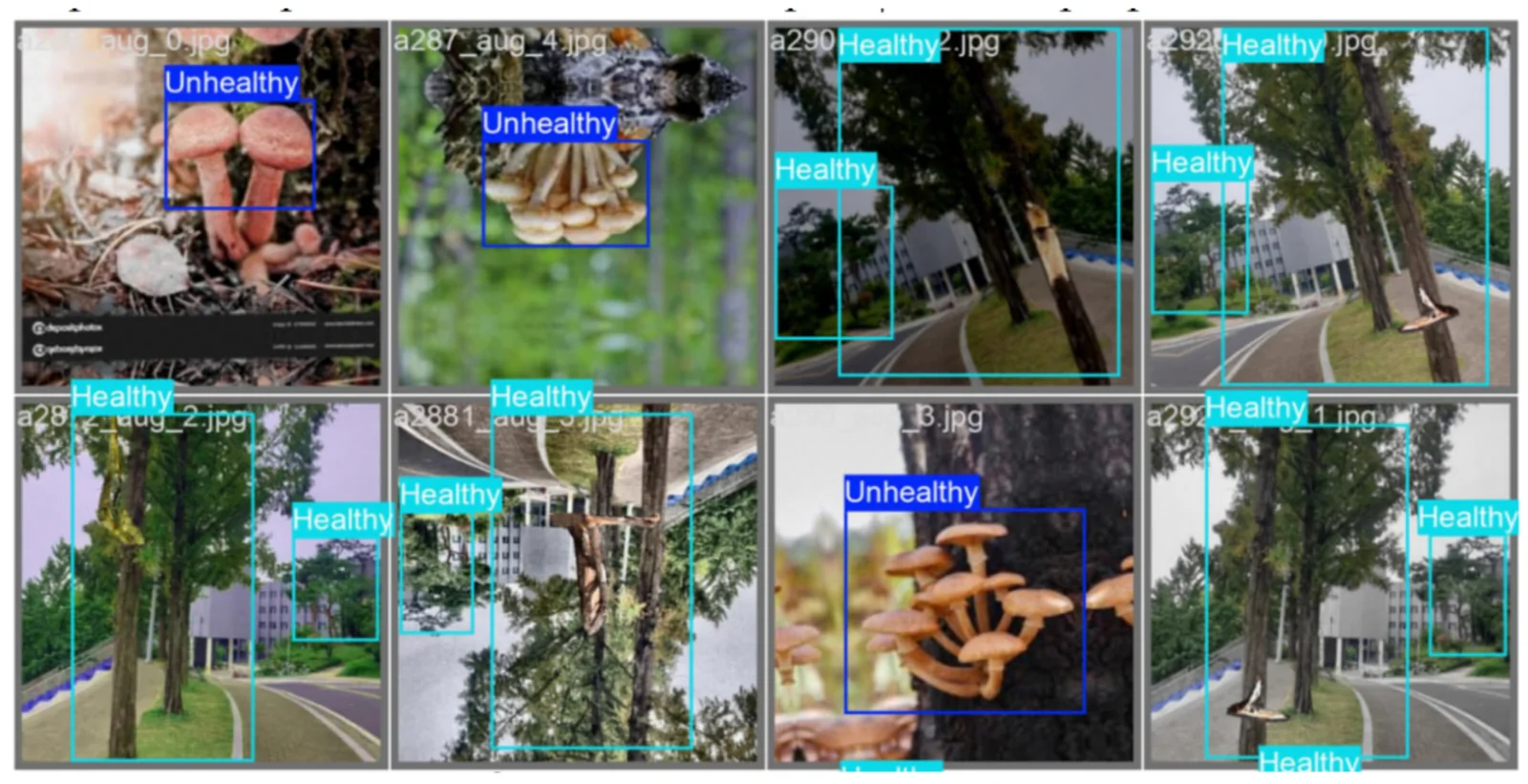
Model training results for validation of Healthy and Unhealthy class-related images.

The proposed attention-enhanced YOLOv11 model was trained for 50 epochs using the customized two-class dataset. Representative qualitative detection results are presented in Figure 8.

The examples illustrate the model’s ability to localize Healthy and Unhealthy targets under varying environmental conditions. Blue bounding boxes denote Unhealthy detections associated with visually observable fungal structures on the tree trunk, root region, or immediate surroundings, whereas cyan bounding boxes denote Healthy detections for which no visually observable fungal structures are identified within the annotated tree region. These qualitative examples complement the quantitative evaluation by illustrating model behavior under variations in background complexity, tree appearance, and fungal-associated visual patterns. The examples demonstrate the model’s ability to localize fungal bodies with clear spatial precision, even under varying illumination, background clutter and perceptive distortions. In Unhealthy samples, the detector accurately identifies fungal clusters attached to tree bark, reflecting the model’s capacity to capture texture-sensitive features enhanced by the SE-like attention module. Conversely, Healthy instances show consistent and stable predictions across multiple viewpoints, suggesting strong separation between non-infected environments and fungus-associated regions.

**Figure 9:**
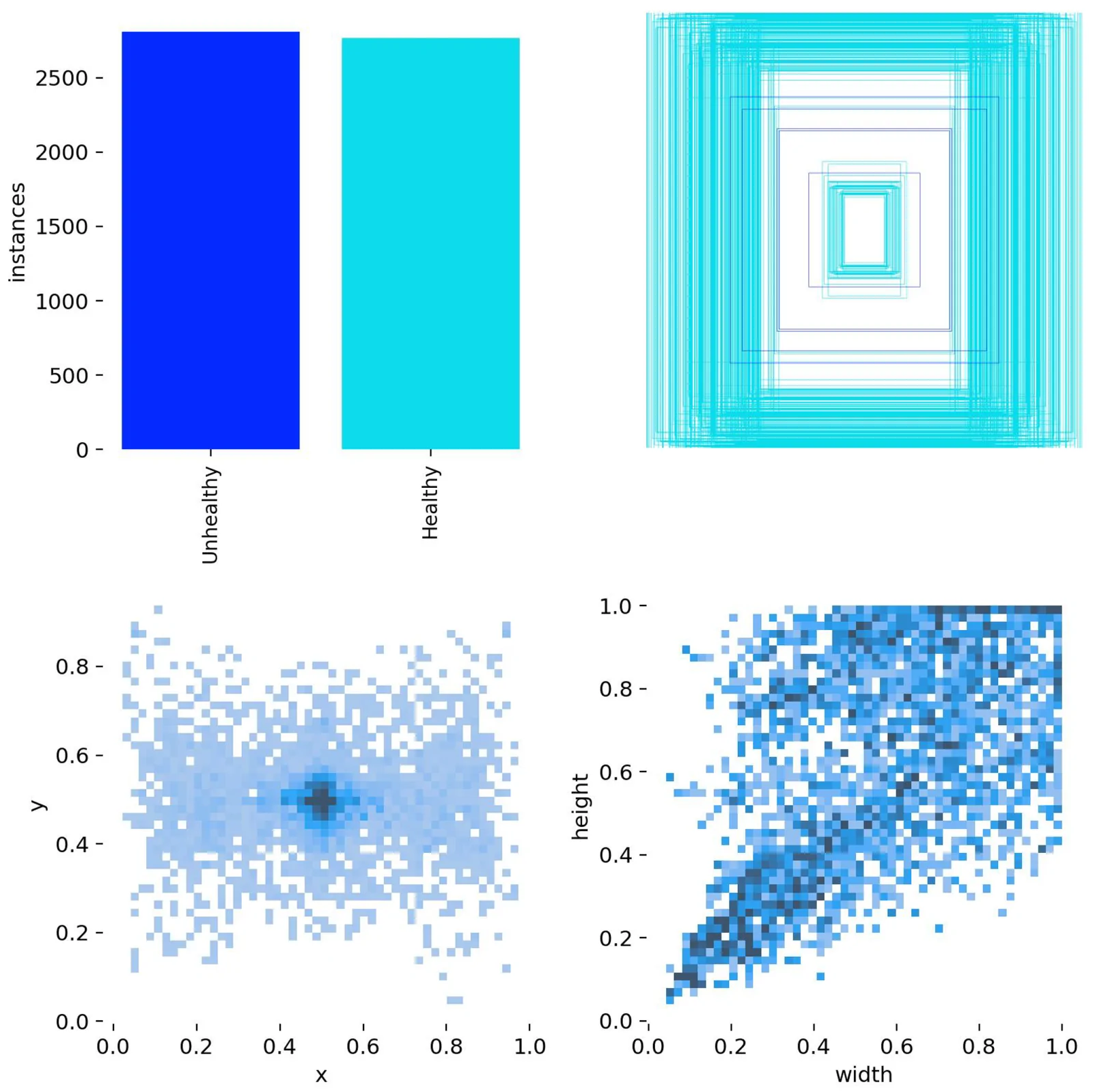
Class distribution and spatial characteristics of annotated bounding boxes in the dataset.

Figure 9 illustrates the class distribution and spatial characteristics of the annotated bounding boxes.

The dataset shows a nearly balanced distribution between Healthy and Unhealthy instances. The bounding-box analysis further presents the normalized spatial distribution of object centers and the variation in bounding-box width and height, indicating considerable diversity in target location and scale across the dataset.

**Figure 10:**
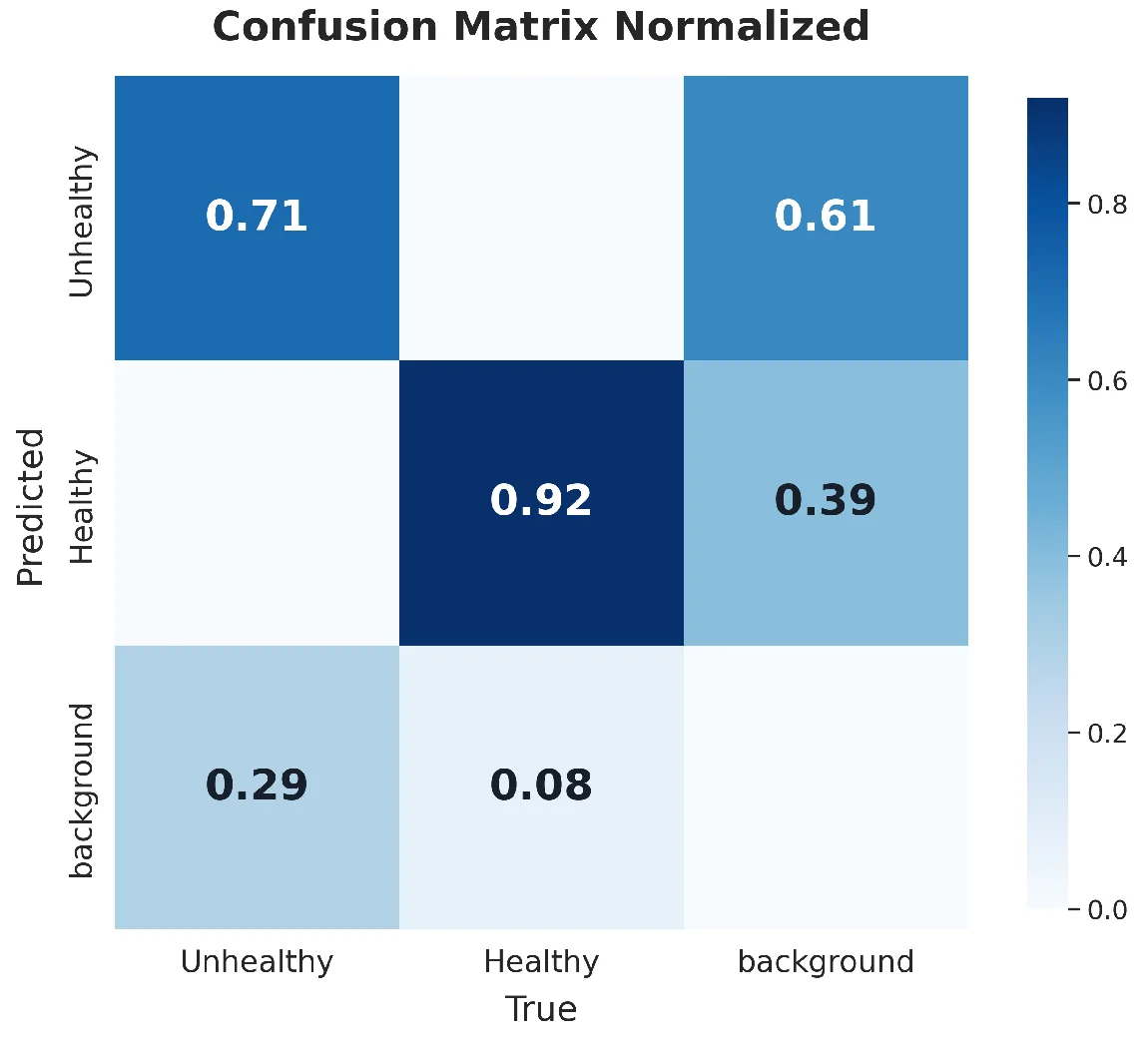
Confusion matrix of the proposed dataset.

The normalized confusion matrix in Figure 10 provides further insight into the class-specific behavior of the proposed model. The Healthy class demonstrates comparatively strong recognition performance, whereas detection of the Unhealthy class remains more challenging, with a recall of approximately 0.71. Notably, the confusion matrix indicates limited direct inter-class confusion between Unhealthy and Healthy predictions. However, the absence of direct Unhealthy-to-Healthy misclassification should not be interpreted as the absence of false negatives. Some ground-truth Unhealthy instances remain undetected and are therefore represented through the background or missed-detection component of the object-detection confusion matrix. This observation is consistent with the lower recall of the Unhealthy class and suggests that subtle fungal structures, low-contrast boundaries, morphological variability, and similarity to bark or surrounding organic materials can result in missed detections. Consequently, improving sensitivity to subtle Unhealthy instances remains an important direction for further model development.

**Figure 11:**
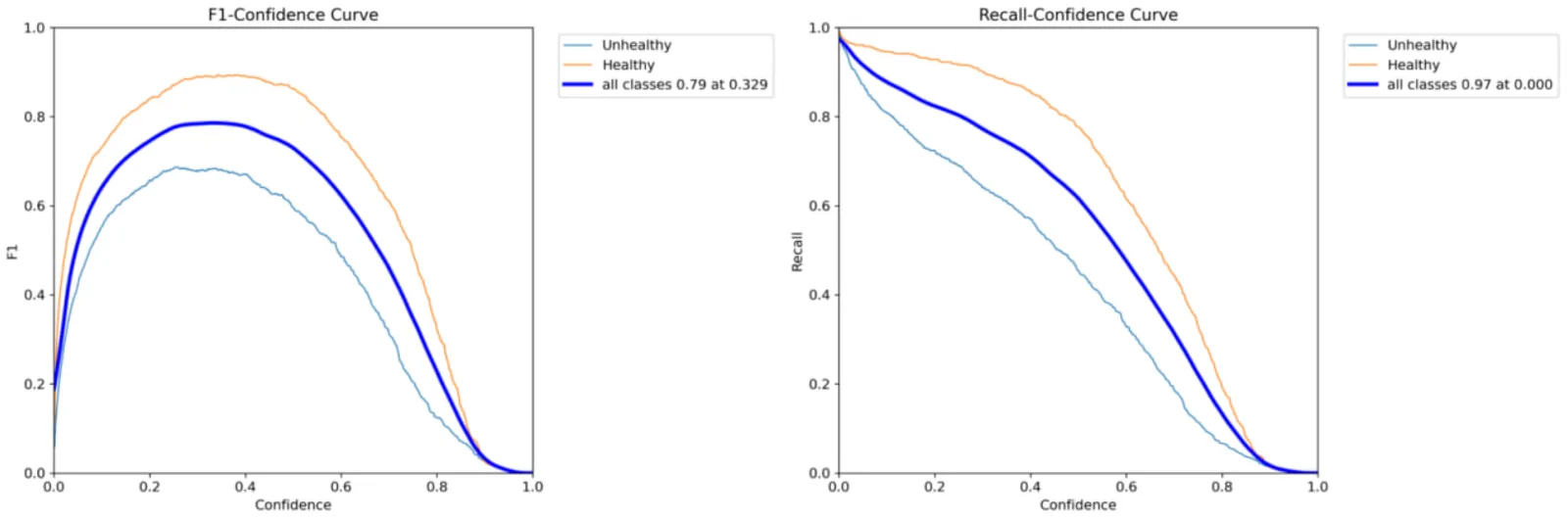
$F_{1}$-Confidence curve (**left**) and Recall-Confidence curve (**right**).

The figure presents F1-Confidence curve and Recall-Confidence curve for our two-class object detection model trained to distinguish between Healthy and Unhealthy trees based on the location or dislocation of fungi nearby trees. The F1-curve (left panel) illustrates the variation in the harmonic mean of precision and recall (F1-score) as a function of the detection confidence threshold (Figure 11).

The F1–Confidence curve demonstrates the relationship between the confidence threshold and the balance between precision and recall. The Healthy class consistently achieves higher F1-scores across most confidence thresholds than the Unhealthy class.

The overall F1-score reaches its maximum value of approximately 0.79 at a confidence threshold of 0.329, indicating that a moderate confidence threshold provides the most favorable balance between false-positive and false-negative detections. The Healthy class reaches a maximum F1-score of approximately 0.88–0.90, whereas the Unhealthy class exhibits a lower peak of approximately 0.67–0.69, indicating comparatively greater difficulty in detecting fungus-associated targets.

At confidence thresholds above approximately 0.6, the F1-score decreases progressively because increasingly restrictive filtering reduces recall and increases the number of missed detections. Conversely, very low confidence thresholds retain a larger number of low-confidence predictions, which may increase false-positive detections and consequently reduce precision. These results indicate that confidence-threshold selection has an important influence on the balance between detection sensitivity and precision.

The Recall–Confidence curve further illustrates the effect of confidence thresholding on detection sensitivity. At very low confidence thresholds, the overall recall approaches approximately 0.97 because most candidate detections are retained. Recall subsequently decreases as the confidence threshold increases. The Healthy class maintains higher recall across most of the evaluated threshold range, whereas the Unhealthy class exhibits a more pronounced decline. This behavior indicates that fungus-associated Unhealthy targets are more likely to be missed when stricter confidence criteria are applied. The observed class-wise difference may be associated with the heterogeneous appearance of fungal structures, their localized nature, and their visual similarity to background features such as bark, soil, and decayed organic material.

Collectively, the F1–Confidence and Recall–Confidence analyses suggest that a confidence threshold of approximately 0.30–0.35 provides a reasonable operating range for the present dataset by balancing precision and recall. For forest-health screening applications, threshold selection should nevertheless be adapted to the intended operational objective, particularly when reducing false-negative detections of fungus-associated targets is prioritized.

**Figure 12:**
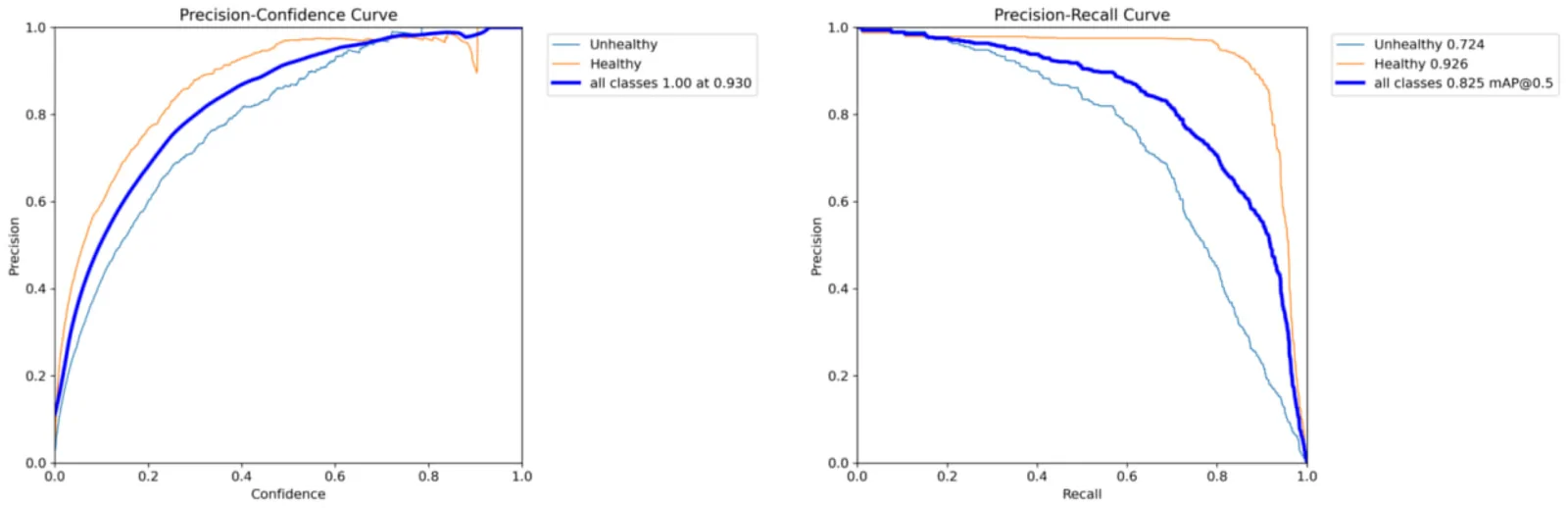
Precision-Confidence curve (**left**) and Precision-Recall curve (**right**).

The model’s detection performance was further evaluated using precision–confidence (P–C) and precision–recall (P–R) curves for the Healthy and Unhealthy target classes (Figure 12). The P–C curve shows that precision generally increases as the confidence threshold becomes more restrictive, reflecting a reduction in low-confidence false-positive detections. Across most confidence thresholds, the Healthy class maintains higher precision than the Unhealthy class, indicating greater reliability in the detection of Healthy targets. At a confidence threshold of approximately 0.93, the aggregated precision reaches 1.00, indicating that predictions retained at this highly restrictive threshold contain very few false-positive detections.

The P–R curves further reveal a clear difference in class-wise detection performance. The Healthy class achieves an AP@0.5 of 0.926, whereas the Unhealthy class achieves an AP@0.5 of 0.724. The overall model achieves an mAP@0.5 of 0.8266. The Healthy-class P–R curve maintains comparatively high precision over a broad range of recall values, indicating more stable detection performance. In contrast, the Unhealthy-class curve exhibits a more pronounced reduction in precision as recall increases, demonstrating the greater difficulty associated with detecting fungus-associated targets. This performance asymmetry may be related to the localized and visually heterogeneous appearance of fungal structures and their similarity to natural background features such as bark, soil, and decayed organic material. Overall, the results presented in Figure 12 demonstrate effective detection performance, while also highlighting the need to further improve sensitivity and precision for the Unhealthy class.

## 5. Discussion

To contextualize the performance and methodological characteristics of the proposed attention-enhanced YOLOv11 framework, recent deep-learning approaches developed for precision forestry and automated plant-disease detection are reviewed. Object detection in heterogeneous forest environments presents substantial challenges, including variable illumination, canopy shadows, complex backgrounds, occlusion, and considerable variation in the appearance and scale of target objects.

Table 7 characterize the computational complexity and deployment efficiency of the evaluated detection architectures at an input resolution of 640×640. Faster R-CNN ResNet-50-FPN is the most computationally demanding model, requiring 41.76 M parameters and 182.5 GFLOPs, followed by RT-DETR-L with 32.97 M parameters and 110.2 GFLOPs. YOLO11n is substantially lighter, with 2.62 M parameters and 6.7 GFLOPs, while the proposed YOLO with SE-like attention has the smallest parameter count at 1.54 M, with a computational cost of 7.7 GFLOPs. This illustrates that model size and computational complexity are distinct characteristics, since the channel-recalibration operations introduced by the SE-like module add computation without substantially increasing the number of learnable parameters. In the CPU-based deployment benchmark, the proposed model achieved the lowest inference latency of 108.80 ms, the highest throughput of 9.19 FPS, and the lowest CPU memory usage among the evaluated models. Although YOLO11n exhibits slightly lower FLOPs, the proposed model provides a favorable overall efficiency profile in terms of parameter count, latency, throughput, and memory consumption. These results indicate that the proposed architecture is suitable for resource-constrained, field-oriented tree-health monitoring; however, broader source-disjoint and real-world field validation remains necessary before making claims of operational generalization.

Table 8 positions the proposed method within representative plant-disease and forestry-related computer-vision research. Prior studies use CNN classifiers, two-stage detectors, YOLO-based one-stage detectors, and transformer-based models for diverse targets, including leaf lesions, fruit, rice symptoms, and canopy-level forest decline. Their reported metrics are presented only as contextual information because the datasets, class definitions, sensors, image scales, and evaluation protocols differ substantially. Unlike the reviewed studies, the proposed model focuses on ground-level detection of fungus-associated unhealthy-tree scenes. Its reported mAP@0.5 of 0.8266 is explicitly identified as exploratory because it was obtained using the original validation split.

The principal demonstrated advantage is its lightweight deployment profile: the model contains 1.54 M parameters and achieved 108.80 ms CPU latency, 9.19 FPS throughput, and 476.45 MB CPU RSS memory. Therefore, the table does not claim numerical superiority over previous methods; rather, it highlights the distinct application setting and efficiency-oriented design of the proposed approach.

The proposed model achieved an overall mAP@0.5 of 0.8266, demonstrating effective detection performance on the developed tree-health dataset. Previous studies have also reported promising results using Faster R-CNN and YOLO-based architectures for agricultural and forest-health applications. For example, Feng et al. [41] employed a YOLO-based framework for pine wilt disease identification, while other studies have investigated two-stage detectors such as Faster R-CNN for plant-disease and lesion detection. However, direct numerical comparison with these studies should be interpreted cautiously because the reported results were obtained using different datasets, target categories, imaging conditions, and evaluation protocols. Therefore, their reported performance values are used primarily to contextualize the proposed approach rather than to claim direct numerical superiority. The present study specifically focuses on ground-level localization of visually observable fungus-associated features under heterogeneous tree, bark, soil, and surrounding environmental conditions.

An SE-like channel-attention mechanism was selected because the visual characteristics relevant to fungus-associated target detection frequently involve subtle differences in texture, color, and local appearance that may be difficult to distinguish from tree bark, soil, leaves, and other organic background structures. The SE-like mechanism performs channel-wise feature recalibration, emphasizing informative feature responses while suppressing less discriminative channels, without introducing extensive spatial processing across the complete feature map. Its relatively lightweight computational structure also makes it suitable for the efficiency-oriented design of the proposed YOLOv11 framework.

The contribution of the attention mechanism was further examined through ablation experiments (Table 9) involving the baseline YOLOv11, a CBAM-enhanced variant, and the proposed SE-like configuration. The three configurations achieved mAP@0.5 values of 0.741, 0.789, and 0.825, respectively. Within this controlled ablation setting, the SE-like configuration achieved the highest mAP@0.5 among the evaluated attention strategies while also maintaining lower inference latency than the CBAM-based alternative. These results provide empirical support for selecting the SE-like mechanism for the final architecture and suggest that lightweight channel recalibration is beneficial for the fungus-associated visual features represented in the developed dataset.

### 5.1. Class-Wise Asymmetry and Practical Epidemiological Value

A detailed assessment of the class-wise predictive behavior using the column-normalized confusion matrix reveals a clear performance disparity between the Healthy and Unhealthy target classes. The Healthy class achieves a true-positive rate of approximately 0.92 and an AP@0.5 of 0.926, indicating comparatively reliable detection. In contrast, the Unhealthy class achieves a lower true-positive rate of approximately 0.71 and an AP@0.5 of 0.724, demonstrating that fungus-associated targets remain more challenging for the proposed model.

This class-wise performance difference is also reflected in the precision–recall analysis, where the Unhealthy class exhibits a more pronounced reduction in precision as recall increases. The reduced performance may be associated with the heterogeneous appearance, irregular spatial distribution, and relatively low visual contrast of externally observable fungal structures, which can resemble surrounding bark, soil, decayed wood, and other organic background features. These characteristics increase the difficulty of consistently localizing fungus-associated targets under heterogeneous field conditions.

Importantly, the confusion matrix indicates that no Unhealthy instances were directly misclassified as Healthy at the evaluated operating point. However, this observation should not be interpreted as the absence of false-negative detections. The Unhealthy-class true-positive rate of approximately 0.71 indicates that a proportion of Unhealthy targets were not successfully detected and were instead associated with the background. From a forest-health screening perspective, these missed detections are particularly important because they may prevent potentially affected trees from being selected for subsequent inspection. Therefore, improving recall for the Unhealthy class remains an important objective for future development of the proposed framework.

### 5.2. Ablation Analysis: Validation of the SE-like Attention Block

To evaluate the contribution of the proposed attention mechanism, an ablation study was conducted by comparing the baseline YOLOv11 architecture with CBAM-based and SE-like attention configurations under the same experimental conditions. The objective was to determine whether channel-wise feature recalibration contributes to improved detection of fungus-associated visual features in heterogeneous tree environments.

As shown in Table 10, the baseline YOLOv11 model achieved an mAP@0.5 of 0.741, while incorporating CBAM increased the mAP@0.5 to 0.789. The proposed SE-like channel-attention configuration achieved the highest mAP@0.5 of 0.825, corresponding to an improvement of 8.4 percentage points over the baseline and 3.6 percentage points over the CBAM variant. Class-wise performance followed a similar trend. For the Healthy class, AP@0.5 increased from 0.834 for the baseline to 0.926 for the proposed configuration, whereas Unhealthy AP@0.5 increased from 0.648 to 0.724. These results indicate that channel-wise feature recalibration contributes positively to the detection performance of the proposed model on the evaluated dataset.

The improvement may be attributed to the ability of the SE-like mechanism to emphasize informative channel responses while suppressing less discriminative features arising from complex background structures. Although CBAM also improved performance relative to the baseline, the SE-like configuration achieved higher AP for both target classes and the highest overall mAP@0.5 among the evaluated configurations. These findings provide empirical support for selecting the SE-like attention mechanism for the final architecture.

The F1–Confidence and Recall–Confidence curves further demonstrate the importance of confidence-threshold selection. The overall F1-score reaches its maximum value of approximately 0.79 at a confidence threshold of 0.329, indicating that a moderate threshold provides a favorable balance between precision and recall for the evaluated dataset. At higher confidence thresholds, recall decreases as increasingly restrictive filtering removes lower-confidence detections, with a more pronounced effect on the Unhealthy class. Based on the observed curves, a confidence range of approximately 0.30–0.35 represents a reasonable operating region when a balanced precision–recall trade-off is desired. However, applications that prioritize the reduction of false-negative Unhealthy detections may require a lower confidence threshold, accepting a corresponding increase in false-positive detections.

## 6. Conclusions

This study developed an attention-enhanced YOLOv11 framework for the automated detection and localization of visually observable fungus-associated tree-health conditions in forest environments. The proposed approach integrates a lightweight SE-like channel-attention mechanism with the YOLOv11 architecture to improve feature discrimination in visually complex scenes containing heterogeneous bark textures, soil, vegetation, and other organic background structures. Rather than providing species-level fungal diagnosis or identifying latent internal colonization, the framework is designed as a non-contact visual screening tool for recognizing externally observable fungal-associated cues that may warrant further tree-health inspection.

Experimental evaluation demonstrated the effectiveness of the proposed framework on the developed two-class dataset. The model achieved a precision of 0.8461, recall of 0.7354, F1-score of 0.7869, mAP@0.5 of 0.8266, and mAP@0.5:0.95 of 0.4739. Class-wise analysis revealed stronger performance for the Healthy target, with an AP@0.5 of 0.926, compared with 0.724 for the Unhealthy target. This performance difference highlights the greater difficulty associated with detecting localized and visually heterogeneous fungus-associated structures under complex environmental conditions.

The ablation analysis further demonstrated the contribution of the proposed attention mechanism. The baseline YOLOv11, CBAM-enhanced variant, and SE-like configuration achieved mAP@0.5 values of 0.741, 0.789, and 0.825, respectively. Thus, the SE-like configuration improved mAP@0.5 by 8.4 percentage points relative to the baseline and by 3.6 percentage points relative to the CBAM configuration under the evaluated experimental conditions. These findings support the use of lightweight channel-wise feature recalibration for enhancing fungus-associated feature representation while maintaining a compact detection architecture. Confidence-threshold analysis additionally showed that the overall F1-score reached its maximum value of approximately 0.79 at a confidence threshold of 0.329. Accordingly, the range of approximately 0.30–0.35 provides a reasonable operating region when a balanced precision–recall trade-off is desired for the present dataset. Applications that prioritize reducing missed Unhealthy detections may require a lower threshold at the cost of additional false-positive detections.

Although the dataset incorporates variations in illumination, bark structure, background complexity, and fungal appearance, it does not fully represent the ecological diversity encountered across different tree species, climatic zones, forest structures, geographic regions, and fungal communities. Consequently, domain shifts associated with previously unseen environmental conditions may reduce detection performance, and the reported results should therefore be interpreted within the distribution represented by the current dataset. Furthermore, RGB-based visual detection is inherently limited to externally observable characteristics and cannot confirm fungal species or detect internal colonization before visible manifestations become apparent.

A limitation of the current annotation scheme is that the two target classes represent different spatial scales: Healthy annotations primarily correspond to tree-level regions, whereas Unhealthy annotations mainly represent localized fungal structures. Therefore, the class-wise AP values should be interpreted according to their respective detection targets rather than as directly comparable Healthy-versus-Unhealthy tree classification accuracies. Future work will investigate a hierarchical approach that independently detects Tree and Fungus instances and subsequently infers tree-health status from their spatial relationships. The proposed RGB-based framework is also limited to externally observable fungus-associated characteristics and cannot detect latent fungal colonization within root, bark, xylem, or phloem tissues before visible manifestations occur. Accordingly, “early detection” in this study refers to identifying visible fungal structures or fungal presence near a host tree before advanced decline symptoms become apparent. The proposed framework should therefore be considered a visual screening tool that complements, rather than replaces, confirmatory approaches such as molecular testing, laboratory analysis, or soil-based assays. Future research will prioritize validation using geographically and ecologically diverse datasets covering additional tree species, fungal communities, climatic conditions, seasons, and forest structures. Source-disjoint and cross-domain evaluation, together with complementary sensing and expert or laboratory confirmation, will be investigated to improve generalizability and support reliable field deployment. 

## Figures and Tables

**Table 1:** Comparison of representative studies on plant disease detection, fungal diagnosis, forest pathology, and attention-enhanced deep learning.

Study	Target	Method	Key Results	Strength	Limitation	Relevance
Li et al. (2024) [25]	Agricultural/ forestry pests	Lightweight Faster R-CNN with MobileNetV3X and attention	5439 and 2183 images; ∼77% fewer parameters and ∼92% fewer FLOPs	Lightweight two-stage detector with complexity analysis	Pest rather than fungal detection; relatively complex two-stage design	Supports attention and lightweight model design
Zhou et al. (2023) [26]	Plant disease recognition	Review of CNN, R-CNN, YOLO, transformers, and domain adaptation	Review study	Identifies scale, background, data, and domain-shift challenges	No specific fungal detector or experimental model	Establishes key challenges in plant-disease detection
Jasalavich et al. (2000) [27]	Internal wood-decay fungi	PCR and molecular analysis	14 basidiomycetes and 25 ascomycetes; detection before measurable wood loss	Early and taxonomically specific internal detection	Requires invasive sampling and laboratory analysis	Shows that latent infection cannot be reliably assessed from RGB imagery alone
Naumann et al. (2005) [28]	Fungi within beech wood	FTIR microscopy and cluster analysis	Localized and discriminated two fungal species	Chemical/spectral discrimination within wood	Requires specialized equipment	Supports multimodal sensing for internal/species-level diagnosis
Gonthier et al. (2015) [29]	Wood-decay fungi in conifers	Multiplex and real-time PCR	∼1 pg DNA detection; 129 naturally infected samples	High sensitivity and taxonomic specificity	Invasive and laboratory dependent	Confirms molecular methods are needed for species-level diagnosis
Jellison and Jasalavich (2000) [30]	Early degradative fungi	Review of molecular/immunological methods	Early-stage fungal DNA detection reported	Detection before advanced deterioration	Laboratory and sampling dependent	Positions RGB detection as complementary visual screening
Deng et al. (2025) [31]	Wood-decay fungi in urban trees	Review of visual, structural, and molecular methods	Review study	Comprehensive multimodal assessment	Many methods require sensors, sampling, or experts	Supports AI as a rapid first-stage screening tool
Zhang et al. (2025) [32]	Fungal succession in decomposing wood	Five-year ecological/microbiome study	Two tree species monitored for five years	Demonstrates temporal fungal-community dynamics	Not a computer-vision study	Supports limitations of purely visual detection
Zhang et al. (2024) [33]	Pine wilt disease, UAV RGB	YOLOv4 + SENet attention	mAP: 79.91%; SENet: P 83.74%, R 69.95%, 49.78 FPS	Channel attention for forest-disease detection	Canopy-level symptomatic detection	Supports SE/SENet-style channel attention in YOLO
Du et al. (2024) [34]	Pine wilt disease, UAV	YOLOv5L-s + ShuffleNetV2 + SimAM	mAP: 95.64%; R: 91.28%; 95.70 FPS	Strong accuracy–speed trade-off	Targets canopy-level symptoms	Supports lightweight attention-enhanced forest monitoring
Zhu et al. (2025) [35]	Grape black-rot lesions	YOLOv8 + SPD-Conv + attention	P: 92.64%; R: 93.28%; AP: 96.17%	Effective small-lesion detection	Leaf-level, visually constrained targets	Supports attention and multi-scale feature extraction
Tomato attention study (2024) [36]	Tomato leaf diseases	YOLOv5m + CBAM/SE/ ECA/CA	Best ECA: mAP@0.5 90.40%; mAP@0.5:0.95 68.80%	Direct comparison of attention mechanisms	Leaf-level controlled application	Shows that attention selection should be task dependent
**Proposed SE-like YOLOv11**	**Ground-level fungus-associated tree screening**	**YOLOv11 + SE-like channel attention**	**P: 0.8461; R: 0.7354; F1: 0.7869; mAP@0.5: 0.8266; mAP@0.5:0.95: 0.4739**	**Lightweight, non-contact localization in heterogeneous scenes**	**Binary screening; RGB-only; no species/internal infection diagnosis**	**Bridges laboratory diagnosis and scalable visual screening**

**Table 2:** Examples of fungal-associated plant diseases and their visually observable manifestations relevant to image-based detection.

Disease/Pathogen	Visible Characteristics	Typical Location	Implication for Vision-Based Detection
Powdery mildew	Grayish or white powder-like fungal growth	Leaves, stems, and fruit	Visually detectable when external growth is present, but appearance may vary with host and illumination
Rust diseases	Reddish, orange, purple, or black spore masses	Leaves, branches, and sometimes trunks	Color cues are potentially informative, although small lesions may be difficult to localize
Anthracnose	Leaf spots, foliar blight, and occasionally cankers	Leaves, twigs, and branches	Large morphological variation across hosts complicates generic detection
Phytophthora root rot	Canopy thinning, dieback, wilting, root discoloration, and trunk cankers	Primarily roots, with secondary above-ground symptoms	Internal/root infection cannot be directly inferred from RGB imagery before external symptoms appear
Armillaria root rot	Mycelial fans, mushrooms at the trunk base, and rhizomorphs	Under bark, roots, and trunk base	External fruiting structures may support visual screening, although infection may precede visible symptoms

**Table 3:** Dataset specifications and construction pipeline.

Attribute	Description
Source and composition	
Data source	Google Images search, filtered by usage rights/Creative Commons license
Scene type	Forestry and tree imagery, including fungal growth on or near tree trunks and root systems
Color space	RGB (3-channel)
Original (pre-augmentation) images	2720
Total images (post-augmentation)	8900
Class distribution	Healthy: 4500 (50.6%)/Unhealthy: 4400 (49.4%)
Dataset split	80% training/20% validation
Training images	7120
Validation images	1780
Annotation	
Annotation protocol	MakeSense AI (open-source annotation platform)
Original annotation format	Polygon (segmentation)
Training annotation format	YOLO bounding box (normalized)—converted from polygon annotations
Synthetic compositing (Unhealthy-class generation)	
Blending method	Alpha compositing (RGBA overlay)
Scale range	10–25% of base tree image width
Placement	Random uniform position within image bounds
Label handling	Fungal crop polygon transformed by matching scale/shift and merged with base tree labels
Base/overlay images	Healthy tree images/isolated fungal-branch crops
Offline dataset augmentation (applied once, before training)	
Library	Albumentations
Transforms	Horizontal flip, brightness/contrast jitter, rotation ($\pm 15^{\circ }$), random scale (±20%)—each p=0.5
Variants per source image	4 (plus original)

**Table 4:** Training configuration and hyperparameters of the attention-enhanced YOLOv11 model.

Parameter	Value
Framework	PyTorch-based Ultralytics YOLO framework
Task	Object detection
Model configuration	my_yolo_attention.yaml
Backbone	YOLOv11 backbone with SE-like attention module
Input image resolution	640×640 pixels
Number of epochs	50
Batch size	8
Optimizer	Auto optimizer selection
Initial learning rate	0.01
Final learning-rate factor	0.01
Momentum	0.937
Weight decay	0.0005
Warm-up epochs	3.0
Warm-up momentum	0.8
Warm-up bias learning rate	0.1
Loss gains	Box: 7.5; Class: 0.5; DFL: 1.5
Validation IoU threshold	0.7
Maximum detections per image	300
Data augmentation	Mosaic: 1.0; horizontal flip: 0.5; translation: 0.1; scale: 0.5; random erasing: 0.4
Color augmentation	HSV-H: 0.015; HSV-S: 0.7; HSV-V: 0.4

**Table 5:** Definitions of the evaluation metrics used in the study.

Metric	Definition
True Positive ($T_{P}$)	Number of positive instances correctly identified by the model.
True Negative ($T_{N}$)	Number of negative instances correctly identified by the model.
False Positive ($F_{P}$)	Number of negative instances incorrectly identified as positive.
False Negative ($F_{N}$)	Number of positive instances that are not correctly detected by the model.

**Table 6:** Overall detection performance of the proposed SE-like YOLOv11 model.

Metric	Value
Precision	0.8461
Recall	0.7354
F1-score	0.7869
mAP@0.5	0.8266
mAP@0.5:0.95	0.4739

**Table 7:** CPU-based deployment efficiency comparison at an input resolution of 640×640 and batch size of one. Latency and throughput are averaged over ten inference runs after three warm-up runs.

Model	Parameters (M)	FLOPs (G)	Latency (ms)	Throughput (FPS)	CPU RSS Memory (MB)
Faster R-CNN ResNet-50-FPN	41.76	182.5	1428.24	0.70	782.57
YOLO11n	2.62	6.7	193.11	5.18	483.89
RT-DETR-L	32.97	110.2	1053.79	0.95	710.44
**Proposed YOLO + SE-like Attention**	**1.54**	**7.7**	**108.80**	**9.19**	**476.45**

Note: Measurements were obtained on an AMD Ryzen Threadripper 1950X CPU. CPU RSS memory denotes post-inference resident memory usage. GPU memory and GPU latency should be measured separately on identical CUDA hardware.

**Table 8:** Comparison of representative plant-disease and forestry-related detection methods. Reported performance values are presented only as contextual information because datasets and evaluation protocols differ.

Study	Method	Target Task	Reported Metric	Parameters	Main Limitation/Advantage
Mohanty et al. [17]	CNN classifier	Plant disease classification	Accuracy >99%	–	Controlled dataset; image-level classification only.
Ferentinos [18]	CNN classifier	Multi-species plant disease diagnosis	Accuracy up to 99.5%	–	No object-level localization.
Duan et al. [25]	Faster R-CNN	Rice leaf symptom detection	mAP ≈0.75	–	Higher computational cost; less suitable for edge deployment.
Fu et al. [39]	YOLOv3-tiny	Kiwifruit detection	mAP ≈0.754	–	Affected by occlusion and dense canopy backgrounds.
Li et al. [40]	YOLOv5	Grape powdery mildew detection	mAP ≈0.812	–	Leaf-level task with limited forest-scene complexity.
Feng et al. [41]	YOLOv8 variant	Pine wilt disease identification	mAP ≈0.870	–	Primarily UAV-based canopy symptom detection.
Carion et al. [21]	DETR	General object detection	AP-based metrics	–	High computational demand and data requirements.
**Proposed model**	**YOLO + SE-like attention**	**Fungus-associated tree-health detection**	**mAP@0.5 = 0.8266**	**1.54 M**	**Lightweight CPU deployment: 108.80 ms latency, 9.19 FPS, and 476.45 MB RSS memory.**

**Table 9:** Performance comparison with recent deep learning implementations in botanical and forestry computer vision tasks.

Methodology/Framework	Target Task and Image Modality	Reported Metric (mAP@0.5)
**Proposed Model**	**Tree Health/Fungi Proximity (Ground-level)**	**0.825**
Feng et al. [41]	Pine Wilt Disease Identification (UAV/Aerial)	0.870
Li et al. [40]	Grape Powdery Mildew Tracking (Leaf-level)	0.812
Fu et al. [39]	Kiwifruit Spatial Localization (Orchard Canopy)	0.754
Duan et al. [25]	Rice Leaf Symptom Segmentation (Two-stage)	0.750

**Table 10:** Architectural ablation results evaluated on the custom two-class tree-health dataset.

Configuration	Attention Module	mAP@0.5	Healthy AP	Unhealthy AP
Baseline	None	0.741	0.834	0.648
Variant A	CBAM	0.789	0.881	0.697
**Proposed**	**SE-like Channel Attention**	**0.825**	**0.926**	**0.724**

## Data Availability

The dataset, associated annotations, and relevant model configuration information supporting the findings of this study are available from the corresponding author upon reasonable request for academic and research purposes. Public redistribution of the complete dataset is subject to the licensing and usage conditions associated with the original image sources.

## Abbreviations

DL	Deep Learning
CNN	Convolutional Neural Network
YOLO	You Only Look Once
R-CNN	Region-based Convolutional Neural Network
mAP	mean Average Precision
FPS	Frames Per Second
GPU	Graphics Processing Unit
BiFPN	Bidirectional Feature Pyramid Network
UAV	Unmanned Aerial Vehicle
PCR	Polymerase Chain Reaction
DNA	Deoxyribonucleic Acid
FTIR	Fourier Transform Infrared
RGB	Red, Green, Blue
LAMP	Loop-Mediated Isothermal Amplification
SE	Squeeze-and-Excitation
CBAM	Convolutional Block Attention Module
ECA	Efficient Channel Attention
CA	Coordinate Attention
SPPF	Spatial Pyramid Pooling Fast
FPN	Feature Pyramid Network
PAN	Path Aggregation Network
IoU	Intersection over Union
CPU	Central Processing Unit
HSV	Hue, Saturation, Value
AP	Average Precision
FLOPs	Floating-Point Operations
RSS	Resident Set Size

## References

[1] Fisher M.C., Henk D.A., Briggs C.J., Brownstein J.S., Madoff L.C., McCraw S.L., Gurr S.J. (2012). Emerging fungal threats to animal, plant and ecosystem health. Nature.

[2] Funke G., von Graevenitz A., E Clarridge J., A Bernard K. (1997). Clinical microbiology of coryneform bacteria. Clin. Microbiol. Rev..

[3] Bebber D.P., Ramotowski M.A.T., Gurr S.J. (2013). Crop pests and pathogens move polewards in a warming world. Nat. Clim. Change.

[4] Lievens B., Brouwer M., Vanachter A.C.R.C., Lévesque C.A., Cammue B.P.A., Thomma B.P.H.J. (2005). Quantitative assessment of phytopathogenic fungi in various substrates using a DNA macroarray. Environ. Microbiol..

[5] Sirinukunwattana K., Raza S.E.A., Tsang Y.-W., Snead D.R.J., Cree I.A., Rajpoot N.M. (2016). Locality sensitive deep learning for detection and classification of nuclei in routine colon cancer histology images. IEEE Trans. Med. Imaging.

[6] Wienert S., Heim D., Saeger K., Stenzinger A., Beil M., Hufnagl P., Dietel M., Denkert C., Klauschen F. (2012). Detection and segmentation of cell nuclei in virtual microscopy images: A minimum-model approach. Sci. Rep..

[7] Drelie Gelasca E., Obara B., Fedorov D., Kvilekval K., Manjunath B.S. (2009). A biosegmentation benchmark for evaluation of bioimage analysis methods. BMC Bioinform..

[8] Paine T.D., Raffa K.F., Harrington T.C. (1997). Interactions among scolytid bark beetles, their associated fungi, and live host conifers. Annu. Rev. Entomol..

[9] Six D.L., Wingfield M.J. (2011). The role of phytopathogenicity in bark beetle–fungus symbioses: A challenge to the classic paradigm. Annu. Rev. Entomol..

[10] Banner F., Tonn S., de Wit J., Ackerveken G.V.D., Berger B., Plett D. (2022). Sensor-based phenotyping of above-ground plant-pathogen interactions. Plant Methods.

[11] https://wri.org.

[12] Curtis P.G., Slay C.M., Harris N.L., Tyukavina A., Hansen M.C. (2018). Classifying drivers of global forest loss. Science.

[13] Hansen M.C., Potapov P.V., Moore R., Hancher M., Turubanova S.A., Tyukavina A., Thau D., Stehman S.V., Goetz S.J., Loveland T.R. (2013). High-resolution global maps of 21st-century forest cover change. Science.

[14] Kowalski T. (2006). Chalara fraxinea sp. nov. associated with dieback of ash (*Fraxinus excelsior*) in Poland. For. Pathol..

[15] Pautasso M., Aas G., Queloz V., Holdenrieder O. (2013). European ash (*Fraxinus excelsior*) dieback—A conservation biology challenge. Biol. Conserv..

[16] McKinney L.V., Nielsen L.R., Hansen J.K., Kjær E.D. (2011). Presence of natural genetic resistance in *Fraxinus excelsior* (Oleraceae) to *Chalara fraxinea* (Ascomycota): An emerging infectious disease. Heredity.

[17] Mohanty S.P., Hughes D.P., Salathé M. (2016). Using deep learning for image-based plant disease detection. Front. Plant Sci..

[18] Ferentinos K.P. (2018). Deep learning models for plant disease detection and diagnosis. Comput. Electron. Agric..

[19] Redmon J., Divvala S., Girshick R., Farhadi A. You only look once: Unified, real-time object detection. Proceedings of the IEEE Conference on Computer Vision and Pattern Recognition.

[20] Ren S., He K., Girshick R., Sun J. (2016). Faster R-CNN: Towards real-time object detection with region proposal networks. IEEE Trans. Pattern Anal. Mach. Intell..

[21] Carion N., Massa F., Synnaeve G., Usunier N., Kirillov A., Zagoruyko S. (2020). End-to-end object detection with transformers. European Conference on Computer Vision.

[22] Barbedo J.G.A. (2019). Plant disease identification from individual lesions and spots using deep learning. Biosyst. Eng..

[23] Phadikar S., Sil J. Rice disease identification using pattern recognition techniques. Proceedings of the 2008 11th International Conference on Computer and Information Technology.

[24] Too E.C., Yujian L., Njuki S., Yingchun L. (2019). A comparative study of fine-tuning deep learning models for plant disease identification. Comput. Electron. Agric..

[25] Li K.-R., Duan L.-J., Deng Y.-J., Liu J.-L., Long C.-F., Zhu X.-H. (2024). Pest Detection Based on Lightweight Locality-Aware Faster R-CNN. Agronomy.

[26] Zhou Z., Zhang Y., Gu Z., Yang S.X. (2023). Deep Learning Approaches for Object Recognition in Plant Diseases: A Review. Intell. Robot..

[27] Jasalavich C.A., Ostrofsky A., Jellison J. (2000). Detection and Identification of Decay Fungi in Spruce Wood by Restriction Fragment Length Polymorphism Analysis of Amplified Genes Encoding rRNA. Appl. Environ. Microbiol..

[28] Naumann A., Navarro-González M., Peddireddi S., Kües U., Polle A. (2005). Fourier Transform Infrared Microscopy and Imaging: Detection of Fungi in Wood. Fungal Genet. Biol..

[29] Gonthier P., Guglielmo F., Sillo F., Giordano L., Garbelotto M. (2014). A molecular diagnostic assay for the detection and identification of wood decay fungi of conifers. For. Pathol..

[30] Jellison J., Jasalavich C.A. (2000). A review of selected methods for the detection of degradative fungi. Int. Biodeterior. Biodegrad..

[31] Deng J., Sun L., Zhang J. (2025). Advance on Investigation and Diagnostic Technology of Wood Decay Fungi in Urban Trees. Subtrop. Plant Sci..

[32] Zhang Y., Peng Z., Song Z., Schilling J.S. (2025). Repeated Measures of Decaying Wood Reveal the Success and Influence of Fungal Wood Endophytes. mSystems.

[33] Zhang Z.B., Han C., Wang X., Li H., Li J., Zeng J., Sun S., Wu W. (2024). Large field-of-view pine wilt disease tree detection based on improved YOLO v4 model with UAV images. Front. Plant Sci..

[34] Du Z.J., Wu S., Wen Q., Zheng X., Lin S., Wu D. (2024). Pine wilt disease detection algorithm based on improved YOLOv5. Front. Plant Sci..

[35] Zhu J.J., Qiu J., Chen S., Chen S., Zhang H. (2025). An application of YOLOv8 integrated with attention mechanisms for detection of grape leaf black rot spots. PLoS ONE.

[36] Lee Y.-S., Patil M.P., Kim J.G., Choi S.S., Seo Y.B., Kim G.-D. (2024). Improved Tomato Leaf Disease Recognition Based on the YOLOv5m with Various Soft Attention Module Combinations. Agriculture.

[37] Zhou G., Whong W.-Z., Marr K.A., Zhong B.-Z., Green F.H.Y., Lewis D., Sorenson W.G. (2000). Development of a fungus-specific PCR assay for detecting low-level fungi in an indoor environment. Mol. Cell. Probes.

[38] Bolikulov F., Abdusalomov A., Nasimov R., Akhmedov F., Cho Y.-I. (2024). Early Poplar (*Populus*) Leaf-Based Disease Detection through Computer Vision, YOLOv8, and Contrast Stretching Technique. Sensors.

[39] Fu L., Feng Y., Wu J., Liu Z., Gao F., Majeed Y., Al-Mallahi A., Zhang Q., Li R., Cui Y. (2021). Fast and accurate detection of kiwifruit in orchard using improved YOLOv3-tiny model. Precis. Agric..

[40] Li L., Zhao H., Liu N. (2023). MCD-Yolov5: Accurate, Real-Time Crop Disease and Pest Identification Approach Using UAVs. Electronics.

[41] Feng X., Zhao X., Shi H., Chen C., Xie Y., Liu M., Xue P. (2025). A feature recognition and detection algorithm for pine wilt disease trees based on FLMP-YOLOv8. PLoS ONE.

[42] Desprez-Loustau M.-L., Robin C., Reynaud G., Déqué M., Badeau V., Piou D., Husson C., Marçais B. (2007). Simulating the effects of a climate-change scenario on the geographical range and activity of forest-pathogenic fungi. Can. J. Plant Pathol..

[43] Yadeta K.A., Thomma B.P.H.J. (2013). The xylem as battleground for plant hosts and vascular wilt pathogens. Front. Plant Sci..

[44] Collins B.R., Parke J.L., Lachenbruch B., Hansen E.M. (2009). The effects of Phytophthora ramorum infection on hydraulic conductivity and tylosis formation in tanoak sapwood. Can. J. For. Res..

